# Identification and Analysis of Small Molecule Inhibitors of CRISPR-Cas9 in Human Cells

**DOI:** 10.3390/cells11223574

**Published:** 2022-11-11

**Authors:** Yue Yang, Donghua Li, Fen Wan, Bohong Chen, Guanglan Wu, Feng Li, Yanliang Ren, Puping Liang, Jian Wan, Zhou Songyang

**Affiliations:** 1 MOE Key Laboratory of Gene Function and Regulation and Guangzhou Key Laboratory of Healthy Aging Research, School of Life Sciences, Sun Yat-sen University, Guangzhou 510275, China; 2 International Cooperation Base of Pesticide and Green Synthesis (Hubei), Key Laboratory of Pesticide & Chemical Biology (CCNU), Ministry of Education, Department of Chemistry, Central China Normal University, Wuhan 430079, China; 3 Key Laboratory of Ophthalmology, Zhongshan Ophthalmic Center, Sun Yat-sen University, Guangzhou 510060, China

**Keywords:** CRISPR, gene-editing, small molecule inhibitor, SpCas9, off-target

## Abstract

Genome editing tools based on CRISPR–Cas systems can repair genetic mutations in situ; however, off-target effects and DNA damage lesions that result from genome editing remain major roadblocks to its full clinical implementation. Protein and chemical inhibitors of CRISPR–Cas systems may reduce off-target effects and DNA damage. Here we describe the identification of several lead chemical inhibitors that could specifically inhibit the activity of *Streptococcus pyogenes* Cas9 (SpCas9). In addition, we obtained derivatives of lead inhibitors that could penetrate the cell membrane and inhibit SpCas9 *in cellulo*. Two of these compounds, SP2 and SP24, were able to improve the specificity of SpCas9 *in cellulo* at low-micromolar concentration. Furthermore, microscale thermophoresis (MST) assays showed that SP24 might inhibit SpCas9 activity by interacting with both the SpCas9 protein and the SpCas9–gRNA ribonucleoprotein complex. Taken together, SP24 is a novel chemical inhibitor of SpCas9 which has the potential to enhance therapies that utilize SpCas9.

## 1. Introduction

Most archaea and 50% of bacteria harbor CRISPR–Cas loci in their genomes [1,2,3,4,5,6,7]. Guided by the CRISPR RNA (crRNA), Cas proteins cleave exogenous DNA/RNA molecules and act as part of the organisms’ adaptive immune system [8,9,10]. The discovery of CRISPR–Cas systems and the subsequent development of genome editing tools have revolutionized genome targeting and manipulation techniques. During genome editing, cleavage by Cas nucleases results in double-stranded DNA breaks (DSBs) [11], which may be repaired through non-homologous end joining (NHEJ) or homology-directed repair (HDR) pathways [12,13,14]. Consequently, off-target cleavage and DNA damage lesions often result from CRISPR–Cas-mediated genome editing and pose significant challenges to its clinical applications. In addition, due to the high activity of the Cas protein, chromosomal translocations and genotoxicity were observed [15]. The Cas protein elicits an immune response [16], and activates the p53 signaling pathway, with a potential cancer risk [17]. Therefore, it is necessary to develop Cas nuclease inhibitors to control the activity of Cas9, thereby improving the safety of CRISPR-based gene therapy. Methods and strategies that help overcome such problems have become areas of intensive research.

About 50 different anti-CRISPR (Acr) proteins have been identified to date, which inhibit different CRISPR–Cas systems [18,19,20] and can be found in the phage genome [21]. Acr proteins inhibit CRISPR-Cas binding or cleavage of target DNA [22]. Despite their effectiveness, Acr proteins may be less than ideal for clinical applications because of their immunogenicity [18,22], slower kinetics, and difficulties in traversing the cell membrane [23]. In addition, large-scale production and storage of Acr proteins can be costly as well. Experimentally, numerous strategies have been devised to increase the specificity and reduce the off-target effects of Cas nucleases. For example, for the most widely studied type II Cas9 nuclease, light-inducible [24,25,26,27] and drug-inducible split-Cas9 systems [28,29,30] were developed to modulate the duration of Cas9 activity; however, the induction kinetics, needed for special instruments, and background activities of such systems have limited their clinical use [31,32]. Small-molecule inhibitors of Cas proteins have also been reported. For example, BRD0539 can inhibit *Streptococcus pyogenes* Cas9 (SpCas9) by blocking its binding to DNA [33], although its IC_50_ is quite high (22 μM). To date, how BRD0539 acts to modulate SpCas9 off-target effects remains unknown [33]. In addition, given the differences in affinity and mode of action of Cas proteins in targeting DNA [34], small-molecule inhibitors that can interact with and/or target Cas9 as well as other Cas proteins in different settings are sorely needed. *In vitro* and *in cellulo* methods to screen and evaluate Cas9 inhibitors are still limited.

In this study, we aimed to identify more specific and high-affinity small-molecule inhibitors of SpCas9. Furthermore, a series of assays were utilized, including *in vitro* cleavage, binding affinity, and cell-based fluorescence screening assays, to better evaluate and study the inhibitors. Through these approaches, we pinpointed several types of lead compound scaffolds that could inhibit SpCas9 *in cellulo*. Two of the derivatives of these lead compounds could effectively reduce SpCas9 off-target activity, thus improving the specificity of SpCas9. We further showed the interaction mechanisms between the inhibitor-derivative compounds and the SpCas9–gRNA complex, which should greatly benefit further explorations of modulating Cas9 activity and specificity.

## 2. Materials and Methods

### 2.1. Cell Culture

Parental and derived HEK293T cells and iCas9 U2OS cells were cultured in Dulbecco’s Modified Eagle’s Medium (DMEM, Corning, New York, NY, USA) containing 10% dialyzed fetal bovine serum (FBS, Biological Industries, Beit HaEmek, Israel) in a humid environment containing 5% CO_2_ at 37 °C.

### 2.2. SpCas9 and dSpCas9 Expression and Purification

The expression and purification of SpCas9 were done as previously described [35]. BL21 Star (DE3)-competent *E. coli* cells (Thermo Fisher, Waltham, MA, USA) were transformed with pET28a-SpCas9 (His6-TEV-NLS tag at N-terminal and the other one NLS tag at C-terminal). A single clone was cultured in LB broth containing 50 μg/mL kanamycin at 37 °C overnight, diluted into 1 L of LB medium with a dilution ratio of 1:100, and cultured at 37 °C until the OD_600_ reached 0.5–0.6. The cells were cooled at 18 °C for 0.5 h and then isopropyl β-D-thiogalactoside (IPTG, Sigma-Aldrich, St. Louis, MO, USA) was added to a final concentration of 0.5 mM. The cells were continued to culture at 18 °C for 14–16 h. The following operations were carried out on the ice or at 4 °C. The cells were centrifuged and resuspended in 10 mL of binding buffer (50 mM Tris-HCl, pH 8.0, 500 mM NaCl, 5% glycerol, and 20 mM imidazole; Sigma-Aldrich) with 50 U benzonase (Sigma-Aldrich, St. Louis, MO, USA). Cells were lysed by sonication and centrifuged at 10,000× *g* for 20 min. The supernatant was filtered with a 0.22 μm PVDF membrane (Millipore, Billerica, MA, USA). The filtered supernatant was loaded into a column with Ni-NTA affinity chromatography column (GE Healthcare, Torrington, CT, USA) and then washed with 20 column volumes of binding buffer. The His-tagged protein was eluted with 10 mL of elution buffer (50 mM Tris-HCl, pH 8.0, 500 mM NaCl, 5% glycerol, and 300 mM imidazole). The eluent was concentrated to approximately 300 μL with Amicon ultra-4 centrifugal filters-30K (Merck Millipore, Billerica, MA, USA) by centrifugation at 4000× *g* and was further purified with a gel-filtration chromatography (HiLoad 10/300 Superdex200, GE Healthcare, Torrington, CT, USA). The protein was eluted with SEC buffer (50 mM Tris-HCl, pH 8.0, 500 mM NaCl, and 5% glycerol) and concentrated. Finally, the protein was filtered with a 0.22 μm PVDF membrane and stored at 4 °C.

### 2.3. Prokaryotic Expression and Purification of LbCas12a

The pET28a-6xHis-TEV-LbCas12a-NLS-3xHA plasmid was transformed into the Rosetta (DE3) *E. coli* strain and protein was induced with 500 μM IPTG at 18 °C for 18 h. Cells were collected and lysed, followed by Ni-NTA affinity chromatography column (GE Healthcare) and Superdex 200 10/300 column (GE Healthcare). The purified proteins were concentrated with Amicon ultra-4 centrifugal filters-50K (Merck Millipore) and quantitated with BCA protein assay Kit (Thermo Fisher Scientific). The proteins were stored at −80 °C.

### 2.4. In Vitro Transcription of crRNA and gRNA

The SpCas9 gRNA and LbCas12a crRNA were *in vitro* transcribed from linear DNA templates using the MEGAshortscript high-yield transcription kit (Invitrogen, Carlsbad, CA, USA) at 37 °C for 4 h. The linearized DNA templates were generated by oligo annealing (IGE Biotechnology Ltd., Guangzhou, China) (Supplementary Table S3). The transcribed RNA was purified with the RNeasy protect mini kit (QIAGEN, Hiden, Germany) and quantified by the Nanodrop 1000 spectrophotometer.

### 2.5. SpCas9 In Vitro Cleavage Assays

The assay was carried out in NEBuffer 3 (New England Biolabs, Ipswich, MA, USA). First, 19.2 μL of the SpCas9–gRNA (58.8 nM) mixture was incubated with 2.4 μL of various compounds (dissolved in DMSO) at desired concentrations (0.1–2 mM) at room temperature for 10 min. Then, 2.4 μL of 3.48 nM ApaI-linearized px458 plasmid was added for further incubation at 37 °C for 60 min. The reaction was terminated by adding 0.5 μL Proteinase K (20 mg/mL) (Invitrogen) and incubation at 55 °C for 15 min. The reaction products were resolved on a 0.7% TAE agarose gel, stained with Gel Red (Biotium, Fremont, CA, USA). Then the products were visualized by Chemidoc XRS plus system (Bio-Rad, Hercules, CA, USA), and band intensities were quantified using ImageJ software (National Institutes of Health developed; Bethesda, MD, USA). The inhibition efficiency in the cleavage assays was calculated using the following equation:

*E* = (*C_1_* − *C_2_*)/*C_1_* × 100%,

where *E* is the inhibition efficiency, and *C_1_* and *C_2_* are the cleavage rate of the DMSO group and compound treated group, respectively.

The cleavage rate was calculated using the following equation:

*C* = (*B_1_* + *B_2_*)/(*B_0_* + *B_1_* + *B_2_*) × 100%,

where *C* is the cleavage rate, *B_0_*, *B_1_*, and *B_2_* are the band intensities of uncleaved products, upper cleaved products, and lower cleaved products, respectively.

### 2.6. LbCas12a In Vitro Cleavage Assays

*In vitro* cleavage experiments were performed using purified recombinant LbCas12a proteins similar to SpCas9 cleavage assays. LbCas12a proteins (50 nM), crRNA (50 nM), and different concentrations of the inhibitor compounds were mixed in NEBuffer 2 (New England Biolabs). After incubation at room temperature for 10 min, ScaI-linearized pUC19 DNA was added to a final concentration of 1.52 nM. The solution was vortex mixed and centrifuged, then incubated for 45 min in a water bath at 37 °C. The reaction was quenched with 0.5 μL proteinase K for 15 min at 56 °C. The samples were mixed with DNA loading buffers and resolved by 1.2% TAE agarose gel electrophoresis. Gel bands were visualized by Chemidoc XRS plus system (Bio-Rad, Hercules, CA, USA).

### 2.7. NotI/XbaI In Vitro Cleavage Assays

*In vitro* cleavage experiments used commercial restriction enzymes (Thermo Fisher Scientific). The restriction enzyme NotI or XbaI (0.1 μL) was mixed with the compounds in a FastDigest buffer (Thermo Fisher Scientific). It was then incubated at room temperature for 10 min, and an ApaI-linearized px458 plasmid was added and reacted at 37 °C for 15 min. The samples were mixed with a DNA loading buffer. The cleavage products were electrophoresed on 1% TAE agarose gel and imaged using a Bio-Rad gel imager.

### 2.8. Establishing the EGFP/iCas9/gRNA-EGFP Reporter 293T Cells

The H2AX-EGFP 293T cell line was first generated using the previously reported MMEJ-mediated CRIS-PITCh system [36,37]. Briefly, we cloned two gRNAs (Supplementary Table S3) that respectively target *H2AX* gene and the PITCh vector into the px330 plasmid (Addgene, #42230). The PITCh vector contains a pair of 20-bp homologous sequences targeting the upstream and downstream regions of the *H2AX* gRNA cleavage site, and the SFB-EGFP expression cassette. The three plasmids were co-transfected into 293T cells at an equimolar ratio. GFP-positive cells were sorted by FACS and individually expanded to isolate single clones for genomic DNA extraction and PCR verification of successful knock-in (KI) of the SFB-EGFP cassette into 5′-end of first exon of the *H2AX* gene. The selected H2AX-EGFP 293T cell clone was subsequently infected with lentiviruses encoding tetracycline-inducible SpCas9 (iCas9). After drug selection, individual clones were again expanded and verified for inducible Cas9 expression. The selected clone was subsequently infected with lentivirus encoding a gRNA that targets EGFP. Puromycin-resistant clones (1 μg/μL, Thermo Fisher Scientific) were then isolated after >7 days for further analysis.

### 2.9. Lentivirus Production

Lentivirus is produced by 293T cells. Specifically, 293T cells were seeded in a 6 cm dish (Thermo Fisher Scientific) before transfection. About 24 h later, the target vector, psPAX2 (Addgene, #12260) and pMD2.G (Addgene, #12259) were transfected together at a ratio of 4:3:1. The virus supernatants were collected 48 h and 72 h after the transient and filtered with a 0.45 μm membrane (Millipore) for usage.

### 2.10. Inhibition of SpCas9 Nuclease Activity in Reporter 293T Cells

The reporter 293T cells were seeded into 96-well plates at a density of 10,000 cells per well and cultured overnight before the addition of DMSO alone or various compounds (in DMSO solution) at appropriate concentrations (0.125–100 μM) with or without 2 μg/mL doxycycline for an additional 72 h of incubation. The cells were then harvested for analysis with the CytoFLEX flow cytometer (Beckman Coulter, Brea, CA, USA) or analyzed by western blot with antibodies as indicated. The results were plotted in the GraphPad Prism 6.0 software or Origin 2020b (OriginLab, Northampton, MA, USA). The inhibiting efficiency in reporter 293T cells was calculated using the following equation:

*E’* = (*P_2_* − *P_1_*)/(*P_0_* − *P_1_*) × 100%,

where *E’* is the inhibiting efficiency in reporter 293T cells, and *P_0_*, *P_1_*, and *P_2_* are the GFP positive cell ratio of the uninduced group, Dox and DMSO treated control group, and Dox and compound treated group, respectively.

### 2.11. High-Throughput Sequencing and Data Analysis

The iCas9-expressing U2OS cells were first through lentiviral infection. The cells were cultured with 1 μg/mL puromycin until all cells in the negative control group were dead. Monoclonal cells were then isolated and verified. Lentiviruses encoding appropriate gRNAs were used to infect the iCas9 U2OS cells, which were further selected with 10 μg/mL Blasticidin S HCL (Gibco, New York, NY, USA) until all cells in the control group were dead. The iCas9/gRNA cells were then plated in 12-well plates (Thermo Fisher Scientific) at ~40% confluence per well at 24 h before the doxycycline (2 μg/mL) addition. An individual inhibitor compound was also added at this point at the appropriate concentrations. DMSO alone served as the control. Cells cultured without doxycycline served as the SpCas9 cleavage control. After 24 h, cells were collected for genomic DNA extraction using the AxyPrep blood genomic DNA miniprep kit (Axygen, New York, NY, USA). Sequences surrounding the on- and off-target sites were amplified using barcoded primers (Supplementary Table S3) and the KOD PCR kit (Toyobo, Japan) to generate the sequencing libraries. Single-read sequencing of the pooled samples were performed on the 150 Hiseq 2000 (Illumina, San Diego, CA, USA). Each experiment was repeated twice. MATLAB was used to split the sequencing file and CRISPResso2 [38] was used to analyze the frequency of indels.

### 2.12. Cytotoxicity Assays

HEK293T reporter cells were plated in 96-well plates (4–6 × 10^3^ cells/well) and cultured for 12 h before the following experiments. The medium was replaced with 100 μL fresh medium containing compounds or DMSO. The medium change was performed 72 h afterward and cytotoxicity was measured by the cell-counting kit-8 (CCK-8, Dojindo Laboratories, Japan) reagent following the manufacturer’s instructions. Graphs were created by GraphPad Prism 6.0 software (San Diego, CA, USA).

### 2.13. Fluorescence Polarization (FP) Analysis

The fluorescence polarization experiment was as previously reported [33]. DNA molecules (sequences are listed in Supplementary Table S3) were synthesized, one oligonucleotide contained 94 bases (3′ end is PAM (NGG) sequence). The DNA was labeled at the 3′ end with fluorescein isothiocyanate (FITC). The other one is complementary to the first 58 bases of the previous oligonucleotide. These two oligonucleotides were annealed to generate the target DNA substrate labeled with fluorescent molecules (FITC-DNA). Unlabeled DNA (PAM-DNA) was considered competitive positive controls. Experiments were performed on black 384-well plates (Corning, New York, NY, USA). 50 nM SpCas9-gRNA (1:1) and different concentrations of the compound were added to the reaction buffer (50 mM Tris-HCl, pH 8.0, 0.1 M NaCl, 10 mM MgCl_2_, and 1 mM DTT). PAM-DNA (1250 nM) was used as a positive control. The mixture was then incubated at room temperature for 30 min, and FITC-DNA was added to the final concentration of 25 nM in each well. Fluorescence polarization signals were measured using a multilabel reader (Victor X5, PerkinElmer, Waltham, MA, USA) within 10 min. These values were plotted using GraphPad Prism 6.0 software (San Diego, CA, USA).

### 2.14. Microscale Thermophoresis (MST) Assays

The MST method was carried out similar to the previous report [34,39]. The binding ability of the compounds to SpCas9 in different states was tested by Monolith NT.115 (NanoTemper Technologies, Munich, Germany). The SpCas9-EGFP fusion protein or the SpCas9-EGFP/gRNA RNP complex (protein: gRNA = 1:1) was diluted with a binding buffer (50 mM Tris-HCl, pH 7.4, 150 mM NaCl, 0.05% Tween-20, and 10 mM MgCl_2_) to make a final concentration of 200 nM. The protein diluent was mixed with the compound diluent in equal volume. The mixture was kept at room temperature for 10 min. Then the sample was loaded into the NanoTemper standard capillaries. All experiments were carried out at 25 °C, 80% MST power, and 50% LED power. The data were analyzed with the NanoTemper MO affinity analysis software (NanoTemper Technologies, Munich, Germany). The raw data were then exported and plotted with Origin 2020b (OriginLab, Northampton, MA, USA).

### 2.15. Molecular Docking

We used Sybyl-X 2.1.1 [40] to conduct the docking study on the interaction region between SpCas9 and compounds. The 3D macromolecules of SpCas9 (PDB: 4ZT9) were obtained from the RCSB Protein Data Bank. All water and ions were removed from the crystal structure, and hydrogen atoms were added. Amber FF99 force field was used for the optimization of the whole protein. The Tripos force field and Gasteiger—Huckel charge were used to optimize the compounds before docking. The Surflex-dock module [41] was used for docking. The highest-scoring conformation was selected, and a 2D plot of virtual hits at SpCas9 binding sites was generated by the Pymol software (version 1.8.x, Schrödinger LLC, New York, NY, USA).

### 2.16. Statistical Analysis

The statistical analysis was carried out using GraphPad Prism 6.0 software (San Diego, CA, USA) to assess differences between the experimental groups. A two-tailed, unpaired *t*-test was used to calculate the p-value. Unless specified otherwise, ns means no significance, significance values are (*) *p* < 0.05, (**) *p* < 0.01, (***) *p* < 0.001, and (****) *p* < 0.0001.

### 2.17. Compound Synthesis and Characterization

See Appendix A for detailed descriptions.

## 3. Results

### 3.1. Identification of SpCas9 Inhibitors through Unbiased Screens

**Figure 1:**
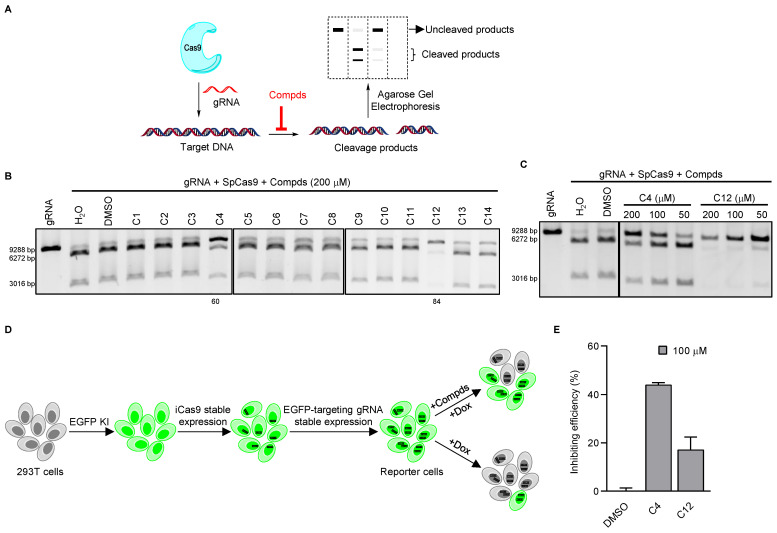
Identification of small-molecule inhibitors of SpCas9 through unbiased screens. (**A**) *In vitro* Cas9 cleavage assays were carried out in the presence of different compounds. Cleavage products were then resolved by agarose gel electrophoresis. (**B**,**C**) SpCas9–gRNA (58.8 nM) mixtures containing various compounds at the indicated concentrations were incubated with a linearized plasmid (3.48 nM) for *in vitro* DNA cleavage assays. The number indicates the compound’s inhibition rate. (**D**,**E**) To monitor the cleavage activity of SpCas9 in live cells, we generated reporter 293T cells where EGFP was knocked into the *H2AX* locus, then tetracycline-inducible Cas9 (iCas9) and an EGFP-targeting gRNA were stably expressed in the cells. The reporter cells were induced with doxycycline (2 μg/mL) in the presence of various compounds (100 μM) and examined for EGFP+ cells by flow cytometry after 72 h (**D**). Results were quantified and plotted in (**E**). Error bars represent SD from two biological repeats.

To identify new small-molecule inhibitors of SpCas9, we chose compounds in our laboratory for initial testing using *in vitro* cleavage assays. These compounds were first grouped based on structural similarities, and we chose compounds containing nitro or hydroxyl groups. Here, successful SpCas9 cleavage of the substrate DNA would result in the appearance of two smaller-sized products via agarose gel electrophoresis (Figure 1A and Supplementary Figure S1A). Of the compounds we tested, C4 and C12 were able to inhibit the activity of SpCas9 at 200 μM concentration with relative efficiencies of 60% and 84%, respectively (Figure 1B). Furthermore, their ability to inhibit SpCas9 was concentration-dependent because the inhibitory effects lessened as their concentrations decreased (Figure 1C). In contrast, the activities of recombinant-bacterial restriction endonucleases XbaI and NotI were not affected by either of these compounds at 200 μM concentration (Supplementary Figure S1B), indicating a specificity toward SpCas9. C4 and C12 are structurally different from each other (Supplementary Figure S1C), suggesting distinct mechanisms of inhibition. To better examine their effects, we established a reporter cell line for the evaluation of SpCas9 activities in live cells. An EGFP tag was first knocked into the 5′-end of the first exon of the *H2AX* gene in 293T cells using the CRIS-PITCh system [36,37] (Supplementary Figure S2A,B). Next, a tetracycline-inducible SpCas9 (iCas9) expression cassette and an EGFP-targeting gRNA were sequentially introduced into these cells to generate the EGFP/iCas9/gRNA-EGFP reporter cell line (Figure 1D). Upon doxycycline (Dox) induction of SpCas9 and subsequent cleavage of the EGFP coding sequence in the *H2AX* locus, the EGFP signal loss could be quantified and serve as an indicator of SpCas9 activity (Figure 1D and Supplementary Figure S2C). In the absence of Dox induction, the two compounds (100 μM) had no effects on EGFP expression in the reporter cells (Supplementary Figure S2D). When we examined cell survival using CCK-8 assays, we found no cytotoxicity in cells cultured with C12 (100 μM) while C4 appeared slightly cytotoxic (Supplementary Figure S2E). Upon induction, more EGFP+ cells could be observed in cells that were also treated with the two compounds, indicating the ability of the two compounds to inhibit SpCas9 activity *in cellulo*. We estimated the efficiency of SpCas9 inhibition at 44.0% and 17.1%, respectively, in this assay (Figure 1E). Overall, our data showed that the two candidate compounds could inhibit the activity of SpCas9 *in vitro* and *in cellulo*.

### 3.2. Exploring the Structure–Activity Relationships of the Candidate Inhibitors

**Figure 2:**
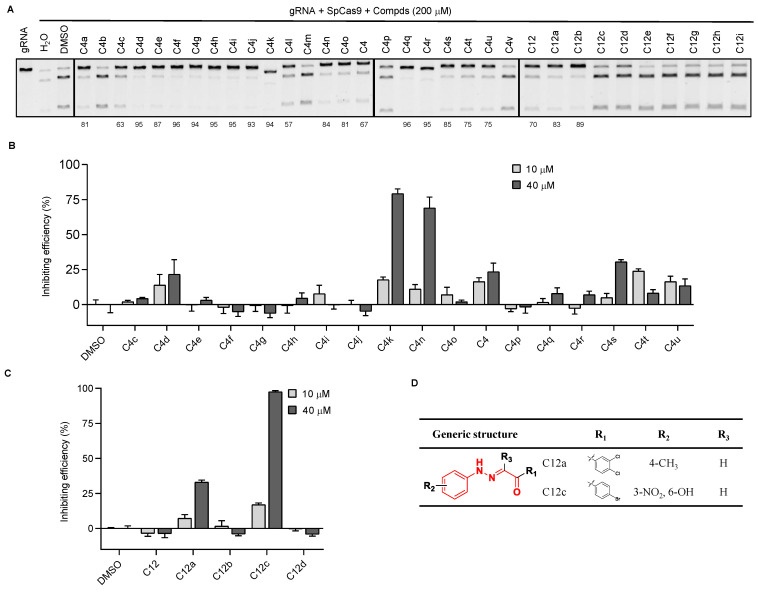
Analysis of structure–activity relationships using analogs of the SpCas9 inhibitors we identified. (**A**) Similar *in vitro* DNA cleavage assays were carried out as described above using the three groups of inhibitor analogs. The number indicates the compound’s inhibition rate. (**B**,**C**) Similar *in cellulo* assessment of the inhibitory activities of the compound analogs was carried out as described above using the reporter 293T cells. Error bars represent SD from three biological repeats. (**D**) The chemical structure of C12a and C12c.

Since the inhibitory activity of C4 and C12 on SpCas9 is not strong, to enhance the activity of these two compounds, we next examined groups of structural analogs of the two compounds in cleavage assays (Supplementary Figure S3A,B). We found that multiple C4 analogs could effectively inhibit the activity of SpCas9 at a 200 μM concentration (Figure 2A). When the activities were correlated with structures (Supplementary Figure S3A), we noticed that the triazole ring in C4 analogs needed to be directly connected to the benzene ring, as in the case of C4a vs. C4b and C4v. Additionally, the inhibitory activity was lost if the chlorine group in the left benzene ring was substituted with a nitro group as in the case of C4a vs. C4m. For C12 derivatives, only C12a and C12b exhibited strong inhibitory activities at 200 μM concentration (Figure 2A). The remaining analogs either lost the activity or only weakly inhibited SpCas9 (e.g., C12c and C12d) in the assays. When the R_1_ position was substituted with phenyl, such as C12 to C12d, the compounds were able to retain some inhibitory activity (Figure 2D and Supplementary Figure S3B), suggesting that the benzene ring at the R_1_ position was critical to the activity of the C12 series of compounds.

We next used the reporter cell line to evaluate the activities of the candidate small-molecule analogs in live cells at much lower concentrations (Figure 2B,C). Of the analogs tested, C4k, C4n, and C12c showed ~50% SpCas9 inhibition at 40 μM, and C12a showed ~33% inhibition. Of these four compounds, C12a and C12c did not negatively affect EGFP expression in uninduced reporter 293T cells (Supplementary Figure S3C) and became the focus of our more in-depth studies.

### 3.3. Compound Optimization Based on Intracellular Activities

**Figure 3:**
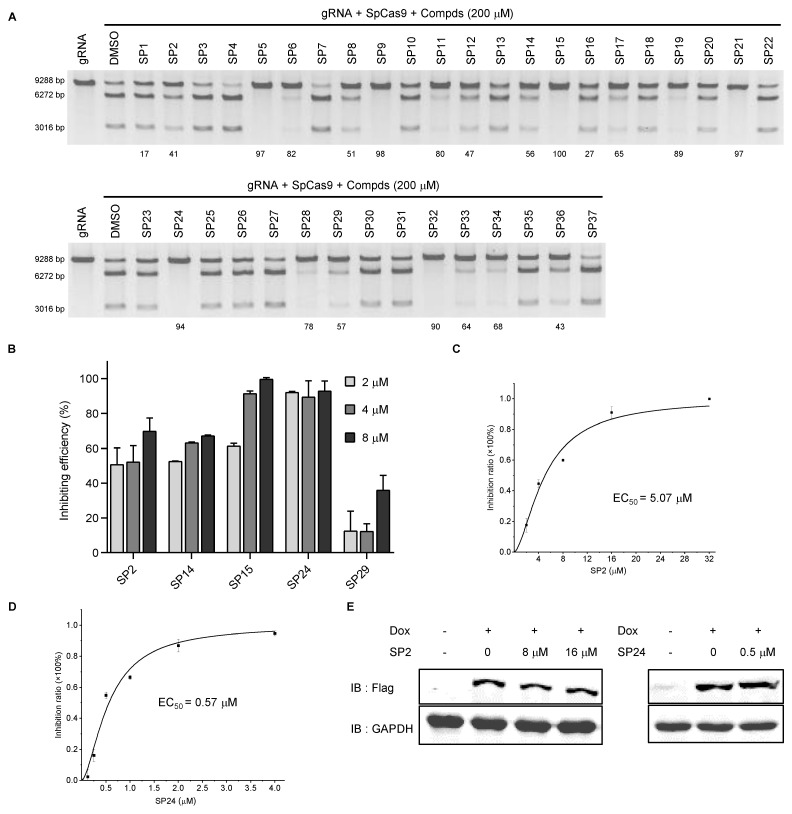
Analysis of *in vitro* and *in cellulo* activities of SP series compounds. (**A**,**B**) The SP series compounds were examined *in vitro* DNA cleavage assays (**A**) and reporter cells (**B**) as above. The number indicates the compound’s inhibition rate. (**C**,**D**) Reporter cells were cultured in Dox (2 μg/mL) plus DMSO or SP2/SP24 at the indicated concentrations for 72 h before flow cytometry analysis. SP2 and SP24 EC_50_ were calculated to be 5.07 μM (**C**) and 0.57 μM (**D**), respectively. Error bars represent SD from three biological repeats. (**E**) Flag-tagged SpCas9 protein expression was determined by western blotting with GAPDH serving as the loading control.

A search of the SPECS website (https://www.specs.net/, assessed on 28 April 2019) using the parent chain of C12a and C12c (Figure 2D) yielded 800+ hits. Because the benzene ring in the R_1_ position is important for the inhibiting activity of the C12 series compounds, we preferentially selected compounds with phenyl at R_1_ position. In addition, in consideration of the diversity of compound structures, we artificially selected some compounds with carbon rings or heterocycles at R_1_ position. Finally, we selected 37 hits from the list (hitherto referred to as the SP series) and determined their *in vitro* and *in cellulo* activities as described above (Supplementary Figure S4 and Supplementary Table S1). At 200 μM concentration, multiple SP series compounds could block *in vitro* SpCas9 cleavage by >50% (Figure 3A). When certain SP compounds were further tested in our CRISPR reporter cells, SP9, SP12, and SP32 showed >50% inhibition at 40 μM concentration; however, these compounds exhibited less than 50% inhibition at 10 μM concentration (Supplementary Figure S5A). The inhibition rate of SP5, SP6, SP8, SP11, SP19, SP28, SP33, SP34, and SP36 were less than 50% at 40 μM concentration (Supplementary Figure S5A). SP2, SP14, SP15, SP24, and SP29 showed 50% inhibition at 10 μM concentration (Supplementary Figure S5A), although four of these compounds (except for SP2) could reduce EGFP expression (albeit slightly) in uninduced cells at 40 μM (Supplementary Figure S5B). Next, we tested the inhibitory activities of these five compounds at even lower concentrations. Among the five, SP15 and SP24 could both inhibit SpCas9 cleavage in cells to a similar extent at 4 and 8 μM concentrations, but only SP24 retained its level of inhibitory activity at 2 μM concentration (Figure 3B). While SP2 appeared not as effective as SP24, its activity changed little from 40 to 2 μM, likely a reflection of the extensive structural differences that exist between the two inhibitors. We calculated the EC_50_ of SP2 and SP24 to be 5.07 μM and 0.57 μM, respectively, through titration experiments using the reporter cells (Figure 3C,D). Neither compound appeared to affect SpCas9 expression at the concentrations tested, supporting a direct inhibition of the SpCas9–gRNA complex (Figure 3E). Importantly, *in vitro* LbCas12a-mediated cleavage assays found no inhibition of LbCas12a by either compound at 100 μM concentration (Supplementary Figure S6), indicating that their activities were specific for SpCas9.

### 3.4. SP2 and SP24 Can Improve the Specificity of SpCas9 in Cells

**Figure 4:**
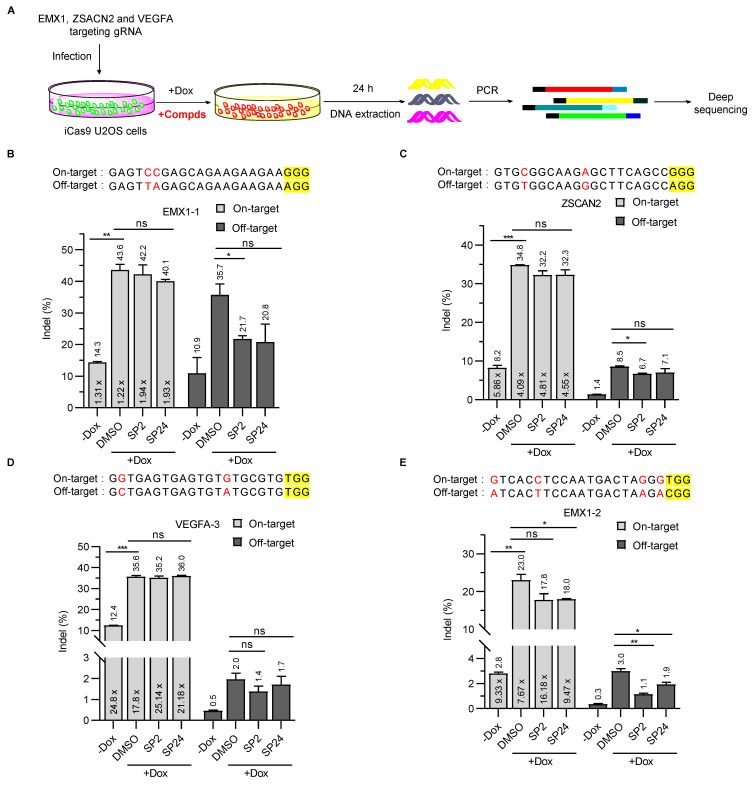
SP2 and SP24 can improve the specificity of SpCas9 in cells. (**A**) To investigate the ability of SP2 and SP24 to inhibit SpCas9 in cells, we established iCas9-expressing U2OS cell lines that also stably expressed gRNAs targeting different endogenous sites. The cells were then cultured with or without Dox and SP2 (2.5 μM)/SP24 (1 μM for VEGFA-3 locus, and 0.5 μM for others) for 24 h before genomic DNA extraction and deep sequencing of on- and off-target sites. (**B**,**E**) The percentage of indels at on- and off-target sites for gRNAs targeting EMX1-1 (**B**), ZSCAN2 (**C**), VEGFA (**D**), and EMX1-2 (**E**) were calculated and plotted as shown. On-target and off-target sites editing efficiency of SpCas9 with SP2 and SP24 treatment at EMX1-1 (**B**), ZSCAN2 (**C**), VEGFA (**D**), EMX1-2 (**E**) locus. Data are from two independent biological replicates. The multiple value in the panel columns was the ratio of on-target vs. off-target indels that were calculated for each condition and target site. PAM sequences are highlighted in yellow. Mismatched bases in on- and off-target sites are marked in red. Error bars indicate mean ± SD. The p values were determined by two-tailed unpaired *t*-test, ns means no significance, (*) *p* < 0.05, (**) *p* < 0.01, and (***) *p* < 0.001.

While the EGFP cDNA had been inserted into the endogenous *H2AX* locus, we wanted to determine whether SP2 and SP24 could effectively inhibit SpCas9-mediated cleavage of *bona fide* endogenous loci. To this end, we first established iCas9-expressing U2OS cells. We then selected four gRNAs that target different sites in three endogenous gene loci and stably expressed these guides in the iCas9 U2OS cells. The cells were subsequently cultured with or without Dox before being collected for DNA extraction and deep sequencing (Figure 4A). Cleavage at all four sites could be detected even in the absence of Dox, an indication of basal SpCas9 expression caused by the leakiness of the tet-on promoter [42,43,44] (Figure 4B–E). As expected, the mutation frequency (indel, %) was low for both on- and off-target cleavage without Dox induction, although considerable variation existed between the four target sites, where EMX1-1 had the highest on- as well as off-targeting levels (Figure 4B). In the absence of inhibitors, Dox-induced Cas9 expression (DMSO alone samples) led to varying degrees of increases in on-site cleavage, from ~3 fold for VEGFA-3 (Figure 4D) to ~10 fold for EMX1-2 (Figure 4E). With inhibitor treatment, the effect on SpCas9 cleavage was also site-dependent. For ZSCAN2 and VEGFA-3, inhibition was minimal with either compound for both on- and off-site editing, with the exception of SP2 on ZSCAN2 off-targeting that showed small but appreciable inhibition (Figure 4C). For EMX1-1, decreases in the average editing efficiency at on-target sites were not significant, but with 2.5 μM SP2 off-target editing decreased from 35.7% to 21.7% (Figure 4B). For EMX1-2, on-site editing decreased from ~23% to ~18% with both compounds, and 2.5 μM SP2 was able to reduce SpCas9 off-target editing by 63.3% (Figure 4E). At all four tested sites, the ratio of on-target to off-target cleavage activity was higher in the uninduced group (-Dox) than in the DMSO-treated induced group (Figure 4B-E), consistent with the idea that SpCas9 may preferentially cleave target sites at low concentrations [45,46,47]. Following Dox induction (+Dox), the cleavage activity ratio of on-target versus off-target sites was consistently higher in the inhibitor treatment groups than the corresponding DMSO control groups (Figure 4B–E), indicating improved specificity due to the presence of SP2 and SP24. Our results thus far indicate that both SP2 and SP24 can inhibit the cleavage activity of SpCas9 in cells, and the extent of their inhibition is highly dependent on the specific genomic sequence context. Although we have proven that SP2 and SP24 can improve the specificity of SpCas9 in U2OS cells, it is necessary to verify the inhibitory activity of compounds on SpCas9 at more endogenous loci in different cell types.

### 3.5. Examining the Inhibitory Mechanisms of SP2 and SP24

**Figure 5:**
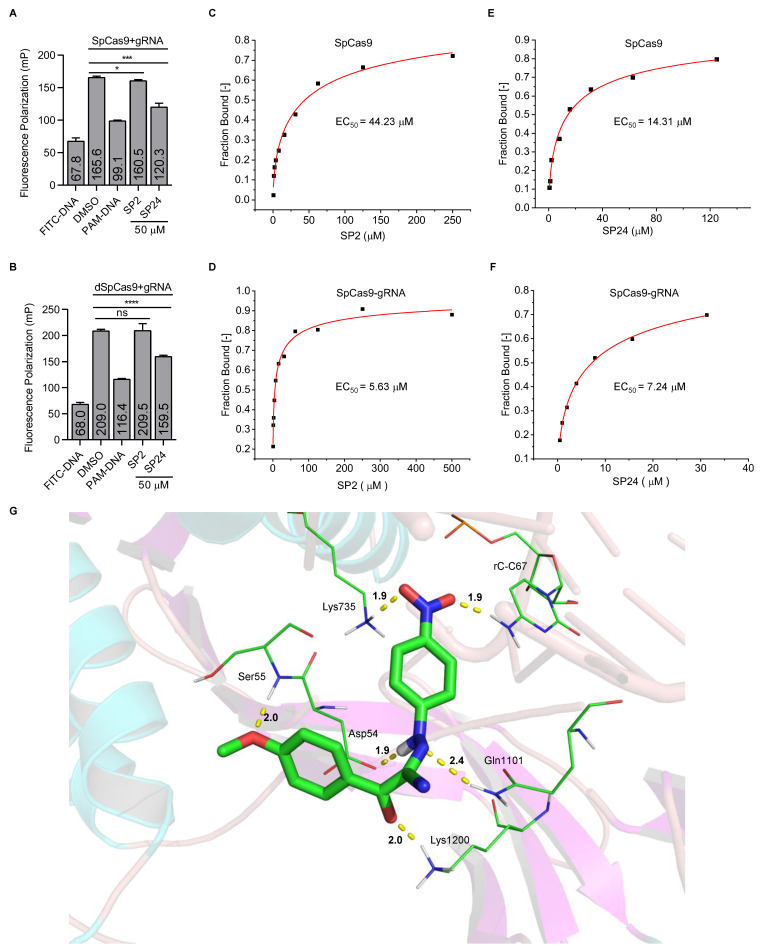
Investigating the inhibition mechanisms of the identified compounds. (**A**,**B**) Each compound (50 nM) was added to a SpCas9-gRNA (1:1) (**A**) or dSpCas9-gRNA (1:1) (**B**) mixture before addition of the FITC-labeled target DNA (25 nM). Fluorescence polarization was assessed by a multilabel reader within 10 min. FITC-DNA, FITC labeled double-stranded target DNA. PAM-DNA, unlabeled double-stranded target DNA (1.25 μM). Error bars represent mean ± SD (n = 3). The p values were determined by two-tailed unpaired *t*-test, ns means no significance, (*) *p* < 0.05, (***) *p* < 0.001, and (****) *p* < 0.0001. (**C**–**F**) For MST assays, the compounds were titrated with SpCas9 or SpCas9-gRNA as indicated. The concentration of SpCas9–EGFP fusion protein was 200 nM. The mixture was kept at room temperature for 10 min. Then the sample was loaded into the NanoTemper standard capillaries. The EC_50_ of SP2 to SpCas9 and SpCas9–gRNA RNP complex is 44.23 ± 35.40 μM (**C**) and 5.63 ± 3.65 μM (**D**), respectively. The EC_50_ of SP24 to SpCas9 and SpCas9–gRNA complex is 14.31 ± 6.9 μM (**E**) and 7.24 ± 3.16 μM (**F**), respectively. (**G**) We propose that SP24 inserts into the active site of SpCas9 (PDB: 4ZT9). The ribbons represent SpCas9 or gRNA. The green rod in the middle represents compound SP24. Amino acid residues or bases that interact with the compound are represented by green lines. The yellow dashed lines represent the hydrogen bond interaction between the compound and the SpCas9–gRNA complex. Carbon, nitrogen, oxygen, and hydrogen atoms are shown in green, blue, red, and white, respectively.

To elucidate the mechanisms underlying the inhibitory activities of SP2 and SP24, we carried out competitive fluorescence polarization (FP) assays, where interaction of the SpCas9–gRNA complex with the fluorescently labeled target DNA could enhance fluorescence polarization signals. As expected, adding unlabeled target DNA (PAM-DNA) to the reaction decreased FP signals (Figure 5A,B). The addition of SP2 at 50 μM did not lead to changes in FP signals of dSpCas9 (nuclease dead SpCas9)-gRNA and DNA (Figure 5B), slightly affect (<5%) FP signals of SpCas9-gRNA, and DNA (Figure 5A), indicating that SP2 may not interfere with the binding of target DNA to the SpCas9–gRNA or dSpCas9–gRNA complex and that SP2 might directly act on SpCas9. In comparison, addition of SP24 led to a significant reduction in FP signals (Figure 5A,B). Compared with DMSO, the addition of SP2 and SP24 did not affect the fluorescence polarization value of fluorescence-labeled DNA (Supplementary Figure S7). Our data suggest that SP24 might function by blocking the interaction between the SpCas9–gRNA complex and the target DNA. To further probe the binding between the compounds and SpCas9, we purified the EGFP-tagged SpCas9 from *E. coil* BL21 (DE3) and carried out microscale thermophoresis assays (MST) (Supplementary Figure S8). We calculated the EC_50_ of SP2 for SpCas9 and SpCas9-gRNA to be 44.23 ± 35.40 μM and 5.63 ± 3.65 μM, respectively (Figure 5C,D). And the EC_50_ of SP24 to SpCas9 and SpCas9–gRNA complex was 14.31 ± 6.9 μM and 7.24 ± 3.16 μM, respectively (Figure 5E,F). These data support a direct interaction between the SpCas9–gRNA complex and the two compounds. Next, we used the SYBYL 2.1.1 Surflex-dock module [40,41] to examine the interaction sites between the SpCas9–gRNA complex (PDB code 4ZT9) and the compounds. Based on molecular docking analysis, SP2 interacts with Tyr72 and Lys65 of SpCas9. In this model, SP2 could form four hydrogen bonds with the gRNA in the SpCas9–gRNA complex, namely cytosine at position 18, guanine at position 19, guanine at position 21, and adenine at position 51 (Supplementary Figure S9). This finding helps explain our observation that the affinity between SP2 and the SpCas9–gRNA complex appeared higher than SP2 and SpCas9. Analysis of SP24 also indicated that the nitro group of SP24 could form hydrogen bonds with the NH_2_ group of Lys735. The NH_2_ group of C67 in the gRNA could also interact with the nitro group of SP24 by hydrogen bonding. And the oxygen atom of the methoxy group in the benzene ring of SP24 could form a hydrogen bond with the hydrogen atom of the peptide bond between Asp54 and Ser55 (Figure 5G). These three hydrogen bonding interactions could lead to the inhibition of SpCas9 by the compounds and helps explain why the inhibitory activity of SP24 appeared greater than that of SP16 and that SP15 was better than SP1 (Figure 3A and Supplementary Figure S4). In addition, the groups between the two benzene rings of SP24 could also form hydrogen bonds with Asp54, Gln1101, and Lys1200 in SpCas9 (Figure 5G). Taken together, our data not only shed light on how SpCas9 activity may be inhibited by small molecules *in cellulo* and *in vitro*, but also help lay the groundwork for further compound modifications and optimization to facilitate the implementation of CRISPR–Cas systems in clinical settings.

## 4. Discussion

In this study, we identified several small-molecule inhibitors of SpCas9, with SP24 showing the highest inhibitory activity *in cellulo*. Our results indicate that SP24 could inhibit the cleavage activity of SpCas9, both in genomically integrated reporter sequences and at *bona fide* endogenous loci. However, whether compounds such as SP24 can effectively inhibit Cas9 nucleases *in vivo* remains to be addressed. Therefore, examining the efficacy and safety of compounds such as SP24 in animal models will be critical to our understanding of how promising inhibitory compounds as well as CRISPR–Cas systems function and interact with each other *in vivo*. We observed that the inhibition rates of the two compounds in 293T reporter cells were higher than those in U2OS cells. Because the rate of GFP degradation will affect the evaluation of inhibition rates, slower GFP degradation may lead to a higher calculated inhibition rate. Therefore, more rapid and sensitive reporter cells are needed to evaluate the inhibitory activity of compounds on SpCas9 in cellulo.

While in-depth studies of inhibitor-Cas9/gRNA interactions can only be possible with high-resolution structures of the complex obtained through crystallography or other means, clues on how chemical compounds such as SP2 and SP24 can achieve effective inhibition can already be gleaned from *in vitro* assays such as FP. For instance, our FP assays showed that SP24 could affect the interaction between the SpCas9–gRNA complex and its target DNA. Further investigation is warranted where residues predicted by molecular docking analysis may be mutated to better pinpoint the residues that are crucial to SP24 action. In addition, we found that SP2 and SP24 significantly inhibit the cleavage activity of SpCas9 at off-target sites while minimally affecting on-target binding, resulting in improved specificity of SpCas9 in U2OS cells. Given the varied inhibitory effects of the compounds on different endogenous target sites, more work is also needed to investigate whether factors such as specific genomic sequences, cell type, and chromatin structure may be determining factors as well. We observed that SP2 and SP24 have little effect on the activity of SpCas9 at endogenous on-target sites, which may depend on the low affinity between the compound and SpCas9. It is necessary to optimize the structure of these compounds and improve the affinity between the compound and SpCas9, to improve the inhibitory activity of these compounds.

The structure of SP24 is different from previously reported small-molecule inhibitors [33,48], and the binding pocket is likely to be different as well. In fact, our data suggest that SpCas9 selectivity could be improved by the compounds. More potent and selective inhibitors of SpCas9 should be attainable based on existing structural information of SpCas9 [49]. We surmise that a cocktail of small-molecule compounds with different structures may be more effective at inhibiting the Cas9 nuclease. In a recent study, SpCas9 variants that could selectively cleave target sites were engineered using a crystal-structure-guided design [49], but the variant is less active in mammalian cells than the wild-type SpCas9 [50]. The mutation sites of the variants are concentrated in the region where SpCas9 interacts with DNA distal from the protospacer adjacent motifs (PAM). Other mutations can improve SpCas9 fidelity by weakening the interaction between SpCas9 and targeted DNA [51,52] or weakening the intermolecular interaction between the domains of SpCas9 [53]. It is possible that mutations in other regions of SpCas9 can also improve SpCas9 its selectivity. Small-molecule inhibitors such as SP2 and SP24 should aid in the identification of such residues/regions on the Cas nucleases. Such endeavors will enable finer control of CRISPR–Cas-mediated cleavage, both its specificity and active time window, in order to reduce the risks of genomic instability and unwanted mutations. In addition, CRISPR–Cas-mediated gene drive has great potential in improving human health, but an uncontrolled gene drive will bring unpredictable risks. Inhibitors of Cas proteins can change the intensity of the gene drive, which is a powerful safety tool [22,54]. Furthermore, routine medical procedures such as blood transfusions, childbirth, and dental surgery may have higher risk of bacterial infection [55,56]. Phage therapy as a means of fighting bacterial infection [55], these small-molecule inhibitors of Cas protein can inhibit bacterial Cas activity and increase the efficacy of bacteriophage therapy [15]. Finally, the inhibitor of Cas proteins can reduce the cytotoxicity caused by the continuous activation of the Cas protein [57].

## Figures and Tables

**Scheme A1:**
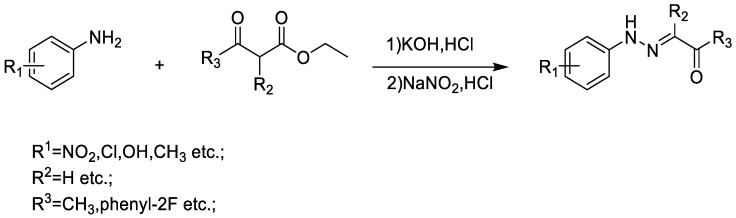
Synthesis pathway of C12 series compounds.

## Data Availability

All raw data of this paper are available upon request. The high-throughput sequencing data from this study have been deposited into the NCBI Sequence Read Archive database (SRA; https://www.ncbi.nlm.nih.gov/sra, assessed on 11 April 2022) under Accession Number NCBI: PRJNA825343 and listed in Supplementary Table S2.

## Supplementary Materials

The following supporting information can be downloaded at: https://www.mdpi.com/article/10.3390/cells11223574/s1, Figures S1 and S2: Related to Figure 1, Identification of small-molecule inhibitors of SpCas9 through unbiased screens; Figure S3: Related to Figure 2. Analysis of structure–activity relationships using analogs of the SpCas9 inhibitors we identified; Figure S4: Related to Figure 3. The chemical structure of SP series compounds; Figure S5: Related to Figure 3. Analysis of SP series compound *in cellulo* activities; Figure S6: *In vitro* LbCas12a cleavage assays were carried out in the presence of different SP series compounds; Figure S7: Fluorescence polarization to detect the interaction between the target DNA and SP2 (or SP24); Figure S8: Prokaryotic expression of SpCas9, dSpCas9, and SpCas9-EGFP; Figure S9: The proposed binding model of SP2 into the active site of SpCas9 (PDB: 4ZT9); Table S1: ID number of SP series compounds in SPECS website; Table S2: List of amplicon sequence data used in this study; Table S3: List of oligos sequence used in this study.

## Appendix A. Compound Synthesis and Characterization

### Appendix A.1. Chemistry

Common reagents and solvents were obtained from commercial suppliers and used without further purification. The compounds were obtained by column chromatography using 200–300 mesh silica gel. The thin-layer chromatography (TLC) with pre-coated glass plates (silica gel 60 GF 254, Qingdao Haiyang Chemical, China) was used to monitor the reaction process. ^1^H and ^13^C spectra were obtained on Varian Mercury-Plus 400 or 600 MHz spectrometers (Bruker, Karlsruhe, Germany). Chemical shifts (δ) were expressed in parts per million (ppm) while the singlet (0 ppm) for tetramethylsilane (TMS). The abbreviations were used to describe peak signal, which s, d, t, q, and m refer to singlet, doublet, triplet, quadruplet, and multiplet, respectively. Coupling constants (*J*) were quoted in HZ. High-resolution mass spectrometry (HRMS) experiments were performed using mass spectrometer with ESI as the ionization.

### Appendix A.2. Synthesis of C4 Series Compounds

The synthesis of C4 series compounds and their detailed characterization will be described elsewhere (Y.Y., manuscript in preparation).

### Appendix A.3. Synthesis of C9 and C12 Series Compounds

The C9 and C12 series compounds were synthesized using the following steps (Scheme A1).

#### General Procedure for the Preparation of C12 Series Compounds

Ethyl acetoacetate (10 mmol) was added into a flask, and then added an aqueous solution of KOH (3.5 g, 100 mL water), and the mixture was stirred at room temperature for 24 h. Once cooled to 0 °C and slowly added concentrated HCl (4.5 mL), the ice water (15 mL) was then added to the mixture. p-nitroaniline(10 mmol) was added to another flask, and then added water (10 mL) and concentrated HCl (3 mL). Once cooled to 0 °C, the mixture was then stirred for 30 min at 0 °C. NaNO_2_ (10 mmol) aqueous solution was added slowly, the temperature should not exceed 5 °C. The mixture was stirred for 30 min to obtain diazonium salt. The freshly prepared diazonium salt was added dropwise to the first flask, and the solution was kept at 0–5 °C. The solution became turbid, and CH_3_COONa aqueous solution (8.2 g, 100 mL ice water) was added dropwise. The mixture was stirred at 0–5 °C for 2 h. The reaction was close to complete, and a yellow solid was obtained by suction filtration.

C9: 1-(2-(4-nitrophenyl) hydrazono)butanone

Orange solid; Yield: 76%; mp: 190.3–191.7 °C; ^1^H NMR (600 MHz, DMSO-*d_6_*) δ 8.18 (d, *J* = 9.2 Hz, 2H), 7.43 (s, 1H), 7.29 (d, *J* = 9.0 Hz, 2H), 2.84 (q, *J* = 6.8 Hz, 2H), 1.04 (t, *J* = 7.3 Hz, 3H). ^13^C NMR (151 MHz, DMSO-*d_6_*) δ 236.86, 187.08, 177.91, 175.53, 163.48, 66.88, 45.73. MS (ESI) *m/z*: 222.0873 [M+H]^+^.

C12: 1-(2-(4-nitrophenyl) hydrazono)-4-bromo-acetophenone

Yellow solid; Yield: 77%; mp: 223.3–224 °C; ^1^H NMR (400 MHz, DMSO-*d_6_*) δ 12.01 (s, 1H), 8.19 (d, *J* = 7.4 Hz, 2H), 7.91 (d, *J* = 8.5 Hz, 2H), 7.77 (d, *J* = 4.7 Hz, 2H), 7.74 (s, 1H), 7.20 (d, *J* = 9.1 Hz, 2H). ^13^C NMR (151 MHz, DMSO-*d_6_*) δ 188.72, 149.23, 141.27, 137.95, 136.02, 131.98, 131.81, 126.96, 126.42, 113.80. MS (ESI) *m/z*: 369.9792 [M+Na]^+^.

C12a: 1-(2-(4-nitrophenyl) hydrazono)-3,4-dichlor-acetophenone

Yellow solid; Yield: 69%; mp: 216.3–218°C; ^1^H NMR (400 MHz, DMSO-*d_6_*) δ 12.08 (s, 1H), 8.20 (d, *J* = 9.2 Hz, 2H), 8.13 (s, 1H), 7.95 (d, *J* = 10.2 Hz, 1H), 7.82 (d, *J* = 8.3 Hz, 1H), 7.76 (s, 1H), 7.20 (d, *J* = 9.1 Hz, 2H). MS (ESI) *m/z*: 335.9957 [M-H]^+^.

C12b: 1-(4-bromophenyl)-2-(2-(4-chlorophenyl)hydrazono)ethanone

Yellow solid; Yield: 65%; mp: 235.2–237.2 °C; ^1^H NMR (400 MHz, DMSO-*d_6_*) δ 11.57 (s, 1H), 7.88 (d, *J* = 8.4 Hz, 2H), 7.71 (d, *J* = 8.5 Hz, 2H), 7.65 (s, 1H), 7.33 (d, *J* = 8.8 Hz, 2H), 7.08 (d, *J* = 8.8 Hz, 2H). ^13^C NMR (151 MHz, DMSO-*d_6_*) δ 188.66, 142.44, 136.57, 134.53, 131.87, 131.63, 129.75, 126.41, 125.74, 115.65. MS (ESI) *m/z*: 358.9592 [M+Na]^+^.

C12c: 1-(4-bromophenyl)-2-(2-(2-hydroxy-5-nitrophenyl)hydrazono)ethanone

Yellow solid; Yield: 76%; mp: 246.6–247.2 °C; ^1^H NMR (600 MHz, DMSO-*d_6_*) δ 11.66 (s, 1H), 11.33 (s, 1H), 8.11 (s, 1H), 7.97 (s, 1H), 7.92 (d, *J* = 8.1 Hz, 1H), 7.76 (t, *J* = 9.9 Hz,2H), 7.01 (d, *J* = 8.8 Hz, 3H). ^13^C NMR (151 MHz, DMSO-*d_6_*) δ 188.96, 151.23, 140.77, 136.64, 136.31, 132.39, 131.87, 131.53, 126.52, 118.57, 115.17, 108.89. MS (ESI) *m/z*: 385.9750 [M+Na]^+^.

C12d: 1-(2,4-difluorophenyl)-2-(2-(4-nitrophenyl)hydrazono)-2-(1H-1,2,4-triazol-1-yl)ethanone

Yellow solid; Yield: 73%; ^1^H NMR (600 MHz, DMSO-*d_6_*) δ 9.36 (s, 1H), 8.53 (d, *J* = 7.7 Hz, 2H), 8.44 (s, 1H), 8.29 (s, 1H), 8.04 (d, *J* = 7.6 Hz, 2H), 7.54 (s,1H), 7.36–7.29 (m, 1H), 7.26 (d, *J* = 9.8 Hz, 1H). ^13^C NMR (151 MHz, DMSO-*d_6_*) δ 182.56, 152.75, 147.82, 146.80, 146.78, 142.32, 132.97, 129.31, 128.43, 125.71, 115.03, 111.90, 111.76, 104.74. MS (ESI) *m/z*: 373.0844 [M+H]^+^.

C12e: 2-(2-(2-hydroxy-5-nitrophenyl)hydrazono)-1-phenylbutane-1,3-dione

Yellow solid; Yield: 70%; ^1^H NMR (600 MHz, DMSO-*d_6_*) δ 12.18 (s, 1H), 7.90 (s, 3H), 7.71 (d, *J* = 6.2 Hz, 1H), 7.68 (s, 1H), 7.53 (d, *J* = 17.8 Hz, 2H), 7.08 (d, *J* = 7.7 Hz, 1H), 2.54 (d, *J* = 9.9 Hz, 3H). ^13^C NMR (151 MHz, DMSO-*d_6_*) δ 198.06, 191.82, 152.48, 140.57, 138.29, 134.29, 132.62, 130.60, 130.37, 128.23, 121.68, 115.90, 110.22, 30.71. MS (ESI) *m/z*: 350.0744 [M+Na]^+^.

C12f: 2-(2-(3-chlorophenyl)hydrazono)-1-(2,4-difluorophenyl)-2-(1H-1,2,4-triazol-1-yl)ethanone

Brown solid; Yield: 60%; ^1^H NMR (600 MHz, DMSO-*d_6_*) δ 11.31 (s, 1H), 8.99 (s, 1H), 8.46 (s, 1H), 7.84 (s, 1H), 7.52 (s, 1H), 7.43–7.27 (m, 2H), 7.19 (s, 1H), 7.12 (d, *J* = 23.3 Hz, 2H). ^13^C NMR (151 MHz, DMSO-*d_6_*) δ 182.85, 153.12, 147.06, 144.02, 134.25, 133.17, 131.58, 128.15, 123.59, 122.99, 115.19, 113.97, 112.01, 104.90. MS (ESI) *m/z*: 236.0613 [M+H]^+^.

C12g: 2-(2-(3-nitrophenyl)hydrazono)-1-phenylbutane-1,3-dione

Yellow solid; Yield: 67%; ^1^H NMR (600 MHz, DMSO-*d_6_*) δ 11.76 (s, 1H), 8.12 (s, 1H), 7.93–7.76 (m, 4H), 7.71 (s, 1H), 7.64–7.52 (m, 3H), 2.54 (s, 3H). ^13^C NMR (151 MHz, DMSO-*d_6_*) δ 196.11, 148.90, 145.64, 140.90, 135.87, 134.83, 131.08, 129.48, 121.21, 116.94, 109.44. MS (ESI) *m/z*: 334.0792 [M+Na]^+^.

C12h: 2-(2-(3-chlorophenyl) hydrazono)-5,5,5-trifluoro-1-phenylpentane-1,3-dione

Gray solid; Yield: 75%; ^1^H NMR (600 MHz, DMSO-*d_6_*) δ 12.34 (s, 1H), 7.77 (d, *J* = 7.8 Hz, 2H), 7.51–7.29 (m, 5H), 7.23 (d, *J* = 7.9 Hz, 1H), 2.42 (s, 2H). ^13^C NMR (151 MHz, DMSO-*d_6_*) δ 188.74, 153.31, 149.01, 144.06, 143.48, 134.50, 131.51, 130.42, 129.52, 127.96, 119.29, 118.70, 21.66. MS (ESI) *m/z*: 391.0430 [M+Na]^+^.

C12i: 1-(2-(2-methoxy-5-nitrophenyl)hydrazono)propan-2-one

Yellow solid; Yield: 79%; ^1^H NMR (600 MHz, DMSO-*d_6_*) δ 8.08–8.06 (m, 1H), 8.05 (s, 2H), 7.82 (s, 1H), 7.59 (d, *J* = 8.8 Hz, 1H), 2.38 (s, 3H), 2.36 (s, 3H). ^13^C NMR (151 MHz, DMSO-*d_6_*) δ 202.15, 152.67, 145.46, 144.96, 131.45, 128.36, 117.90, 108.56, 29.46, 22.71. MS (ESI) *m/z*: 224.0687 [M+Na]^+^.
