# Supplementary material for: Identification and Analysis of Small Molecule Inhibitors of CRISPR-Cas9 in Human Cells
Source: Cells. 2022 Nov 11;11(22):3574. doi: 10.3390/cells11223574 (PMC9688475; doi:10.3390/cells11223574)
Supplement: Supplementary file 1 [file cells-11-03574-s001.zip › Supplementary Figures.pptx]

## Slide 1
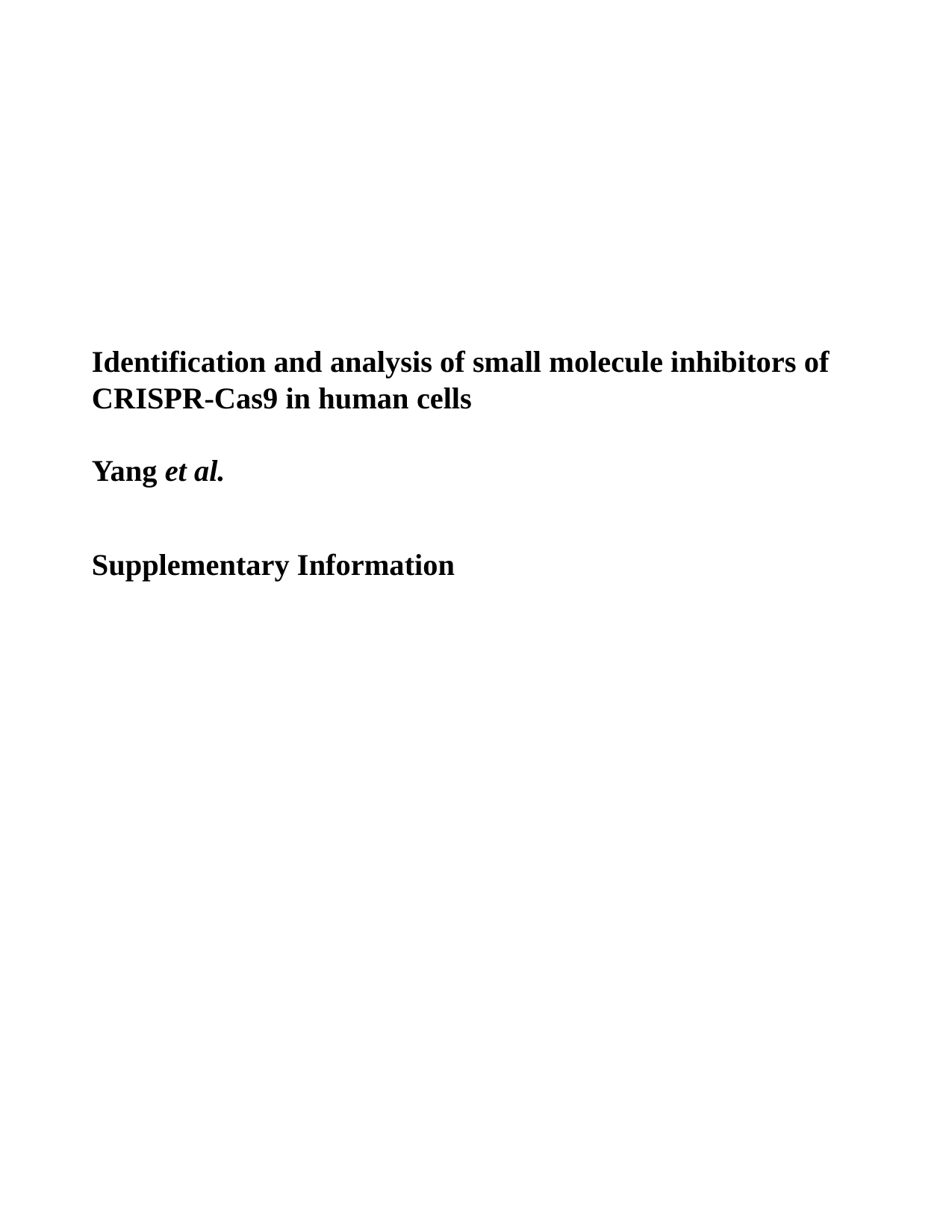

Identification and analysis of small molecule inhibitors of CRISPR-Cas9 in human cells
Yang et al.
Supplementary Information

## Slide 2
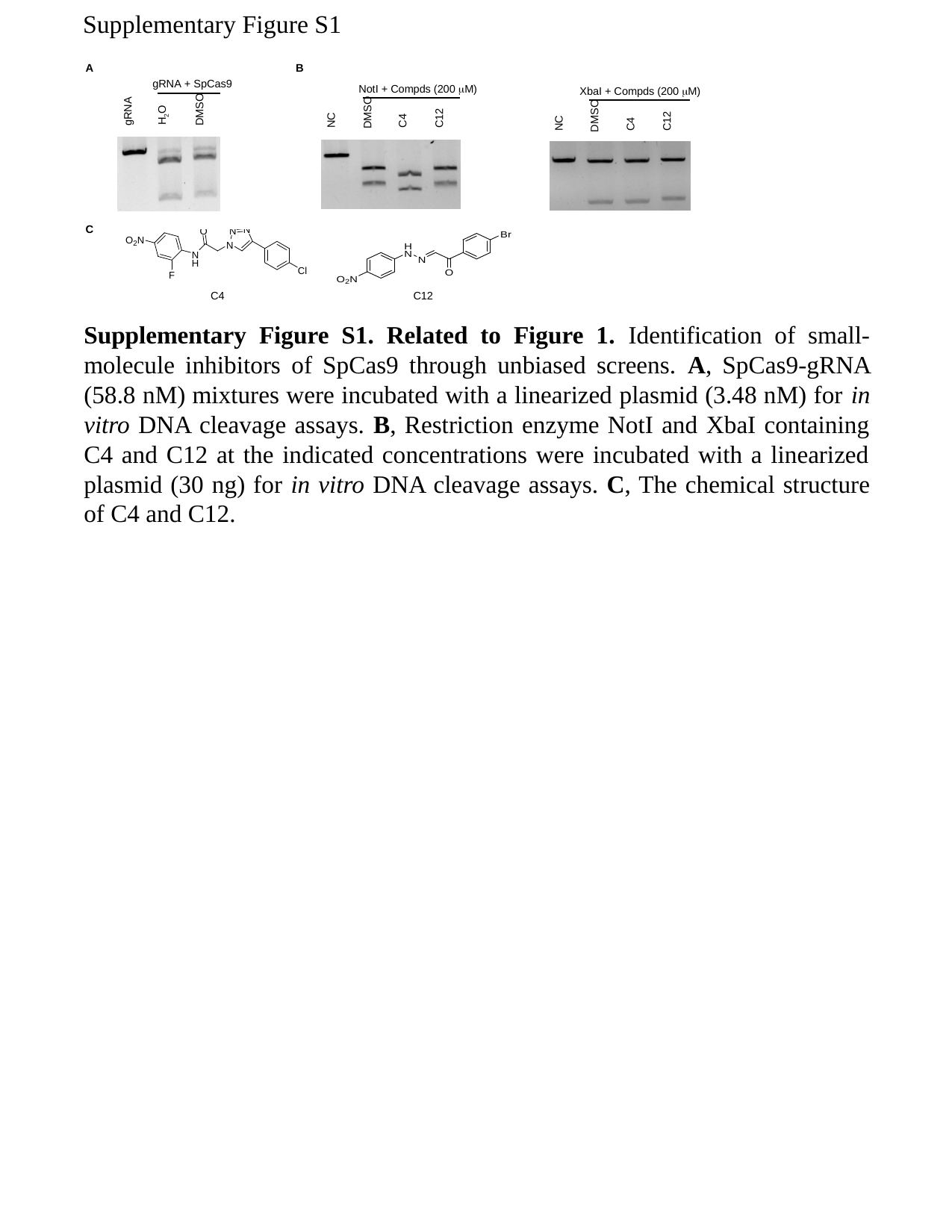

Supplementary Figure S1
A
B
gRNA + SpCas9
DMSO
gRNA
H2O
NotI + Compds (200 mM)
XbaI + Compds (200 mM)
DMSO
DMSO
C12
C12
NC
C4
NC
C4
C
C4
C12
Supplementary Figure S1. Related to Figure 1. Identification of small-molecule inhibitors of SpCas9 through unbiased screens. A, SpCas9-gRNA (58.8 nM) mixtures were incubated with a linearized plasmid (3.48 nM) for in vitro DNA cleavage assays. B, Restriction enzyme NotI and XbaI containing C4 and C12 at the indicated concentrations were incubated with a linearized plasmid (30 ng) for in vitro DNA cleavage assays. C, The chemical structure of C4 and C12.

## Slide 3
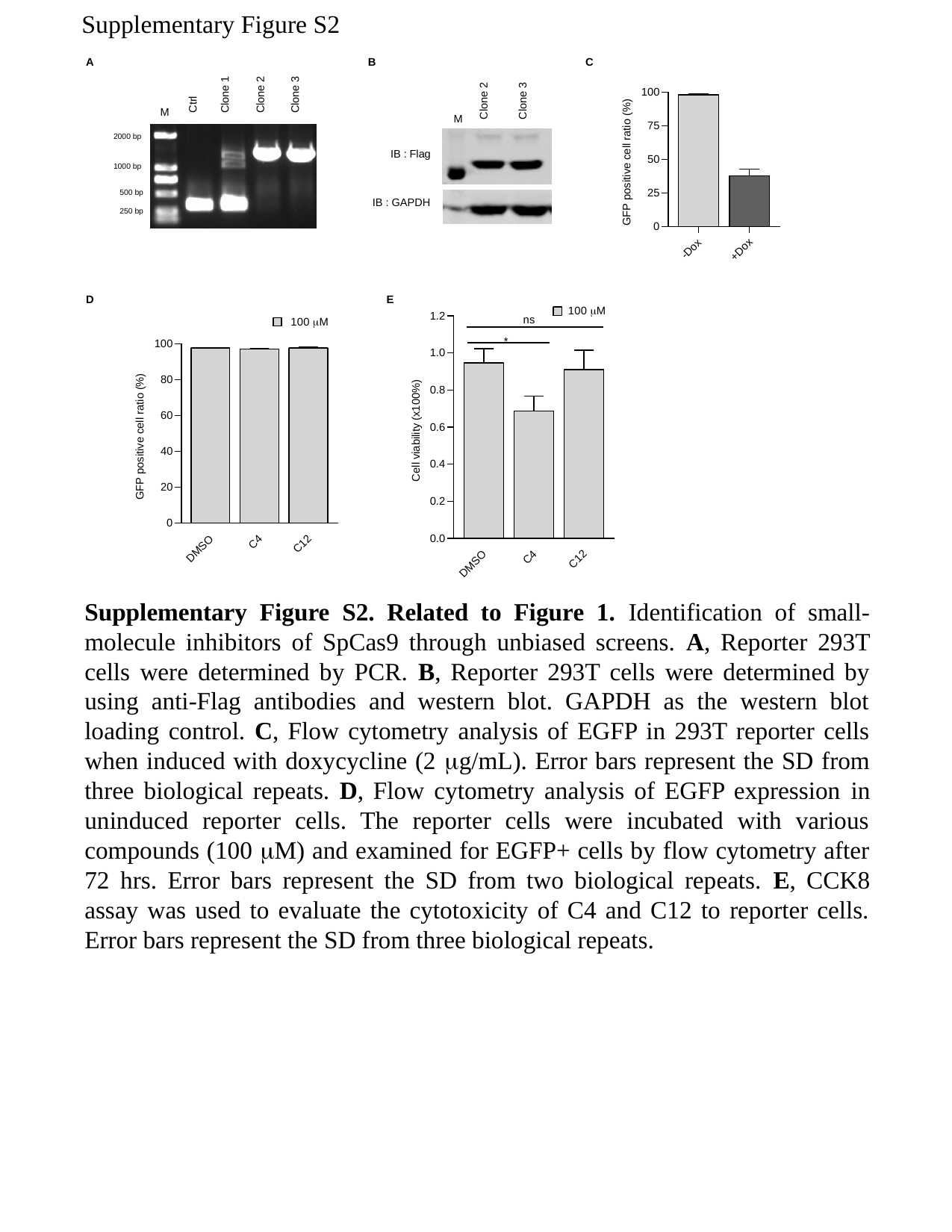

Supplementary Figure S2
A
B
C
Clone 1
Clone 2
Clone 3
Ctrl
M
2000 bp
1000 bp
500 bp
250 bp
Clone 2
Clone 3
M
IB : Flag
IB : GAPDH
D
E
Supplementary Figure S2. Related to Figure 1. Identification of small-molecule inhibitors of SpCas9 through unbiased screens. A, Reporter 293T cells were determined by PCR. B, Reporter 293T cells were determined by using anti-Flag antibodies and western blot. GAPDH as the western blot loading control. C, Flow cytometry analysis of EGFP in 293T reporter cells when induced with doxycycline (2 mg/mL). Error bars represent the SD from three biological repeats. D, Flow cytometry analysis of EGFP expression in uninduced reporter cells. The reporter cells were incubated with various compounds (100 mM) and examined for EGFP+ cells by flow cytometry after 72 hrs. Error bars represent the SD from two biological repeats. E, CCK8 assay was used to evaluate the cytotoxicity of C4 and C12 to reporter cells. Error bars represent the SD from three biological repeats.

## Slide 4
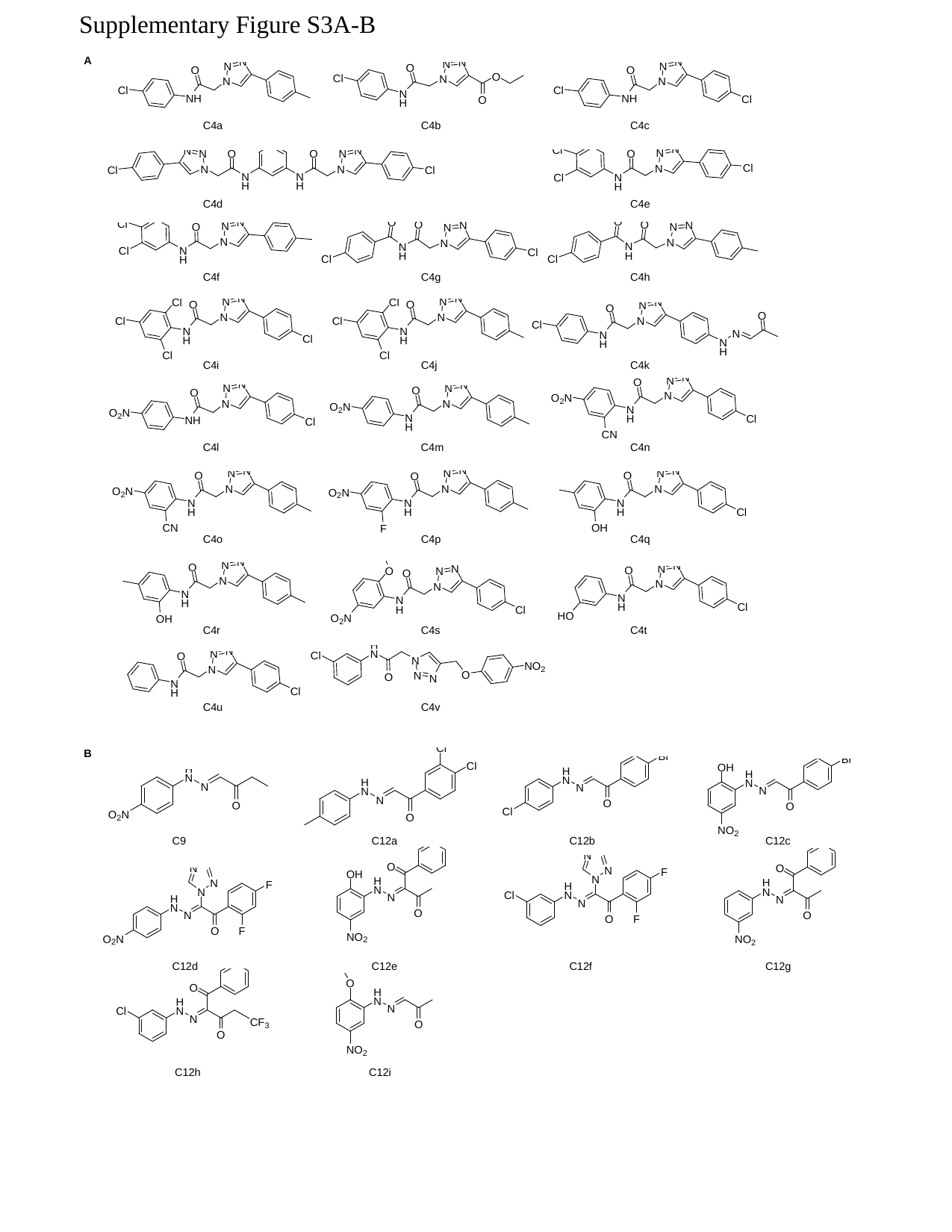

Supplementary Figure S3A-B
A
C4a
C4b
C4c
C4d
C4e
C4f
C4g
C4h
C4i
C4j
C4k
C4l
C4m
C4n
C4o
C4p
C4q
C4r
C4s
C4t
C4u
C4v
B
C9
C12a
C12b
C12c
C12d
C12e
C12f
C12g
C12h
C12i

## Slide 5
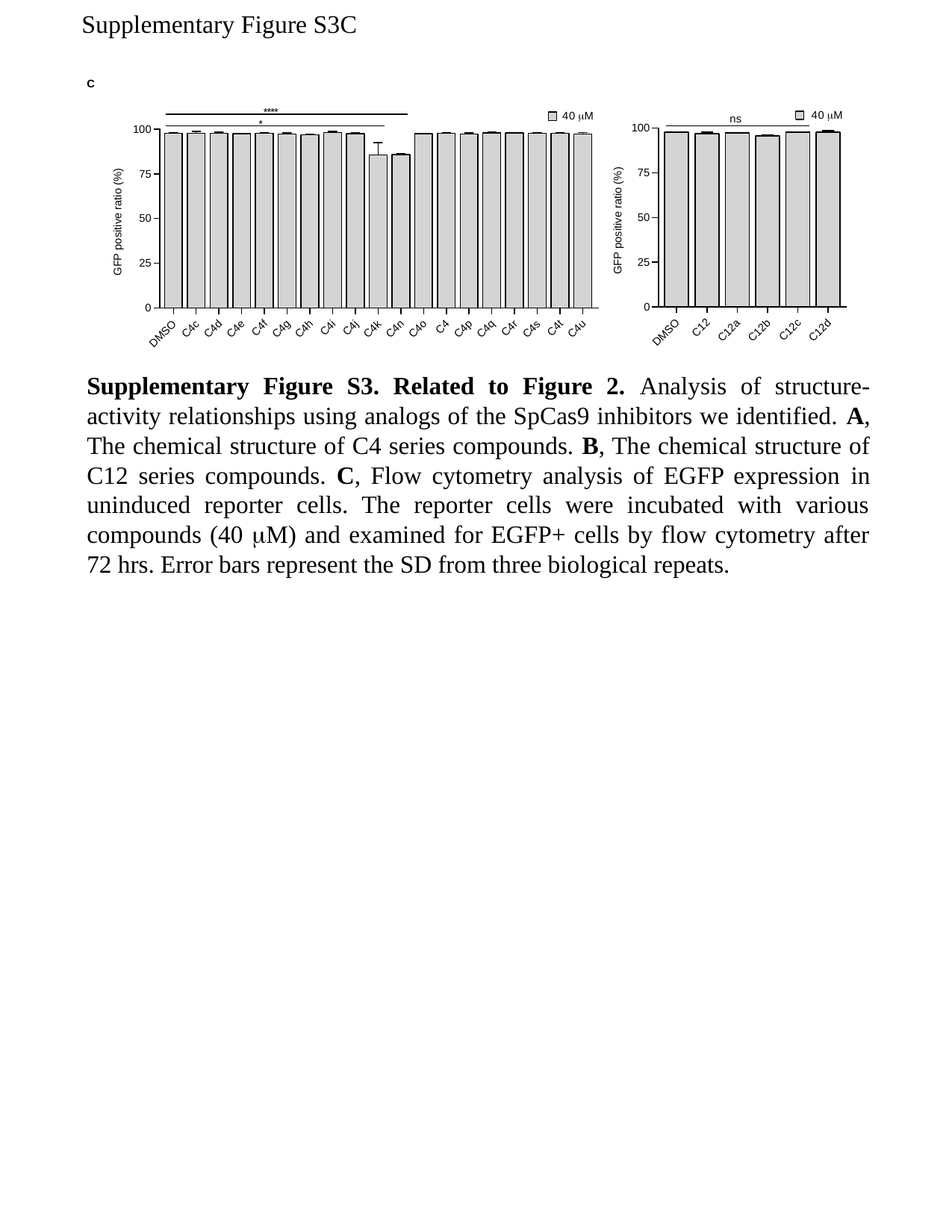

Supplementary Figure S3C
C
Supplementary Figure S3. Related to Figure 2. Analysis of structure-activity relationships using analogs of the SpCas9 inhibitors we identified. A, The chemical structure of C4 series compounds. B, The chemical structure of C12 series compounds. C, Flow cytometry analysis of EGFP expression in uninduced reporter cells. The reporter cells were incubated with various compounds (40 mM) and examined for EGFP+ cells by flow cytometry after 72 hrs. Error bars represent the SD from three biological repeats.

## Slide 6
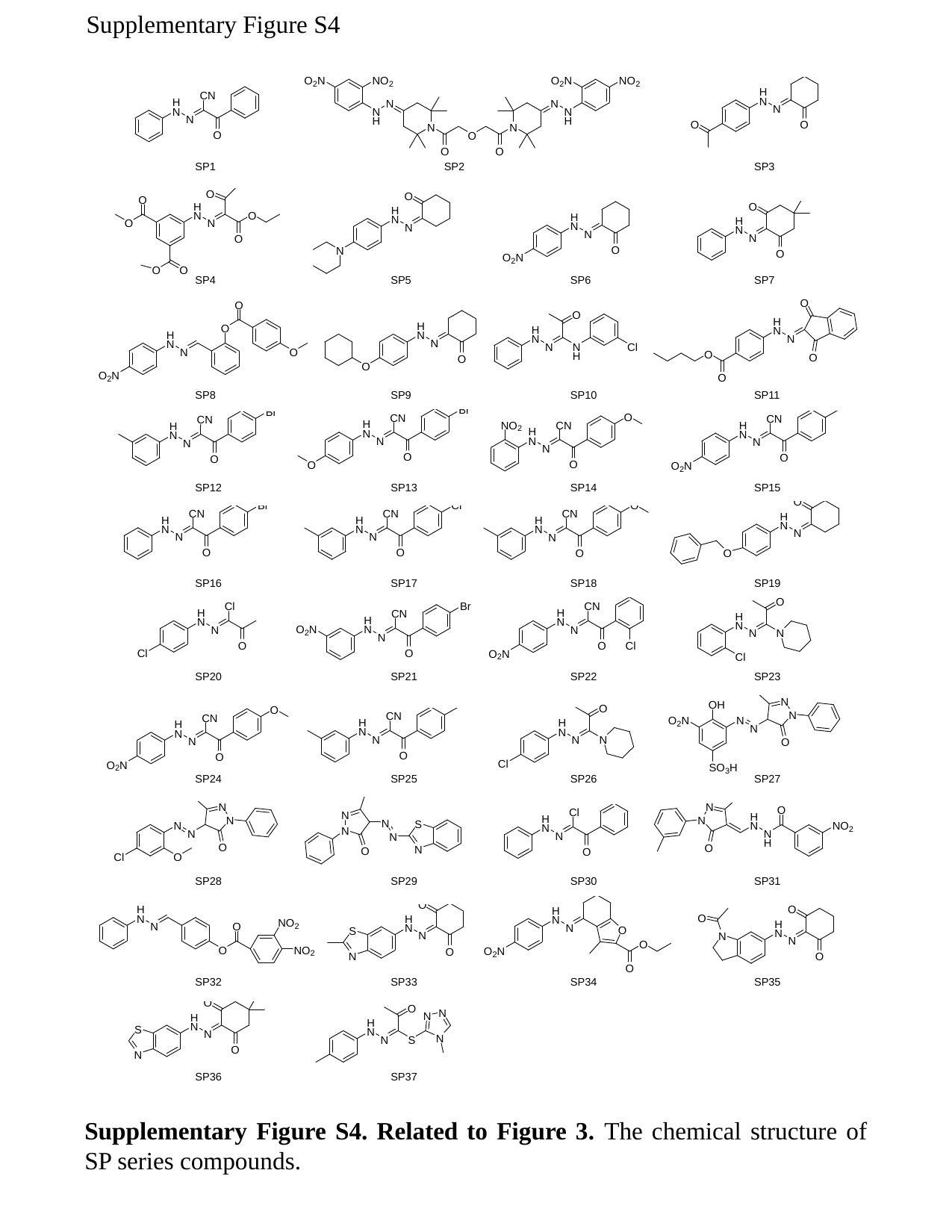

Supplementary Figure S4
SP1
SP2
SP3
SP4
SP5
SP6
SP7
SP8
SP9
SP10
SP11
SP12
SP13
SP14
SP15
SP16
SP17
SP18
SP19
SP20
SP21
SP22
SP23
SP24
SP25
SP26
SP27
SP28
SP29
SP30
SP31
SP32
SP33
SP34
SP35
SP36
SP37
Supplementary Figure S4. Related to Figure 3. The chemical structure of SP series compounds.

## Slide 7
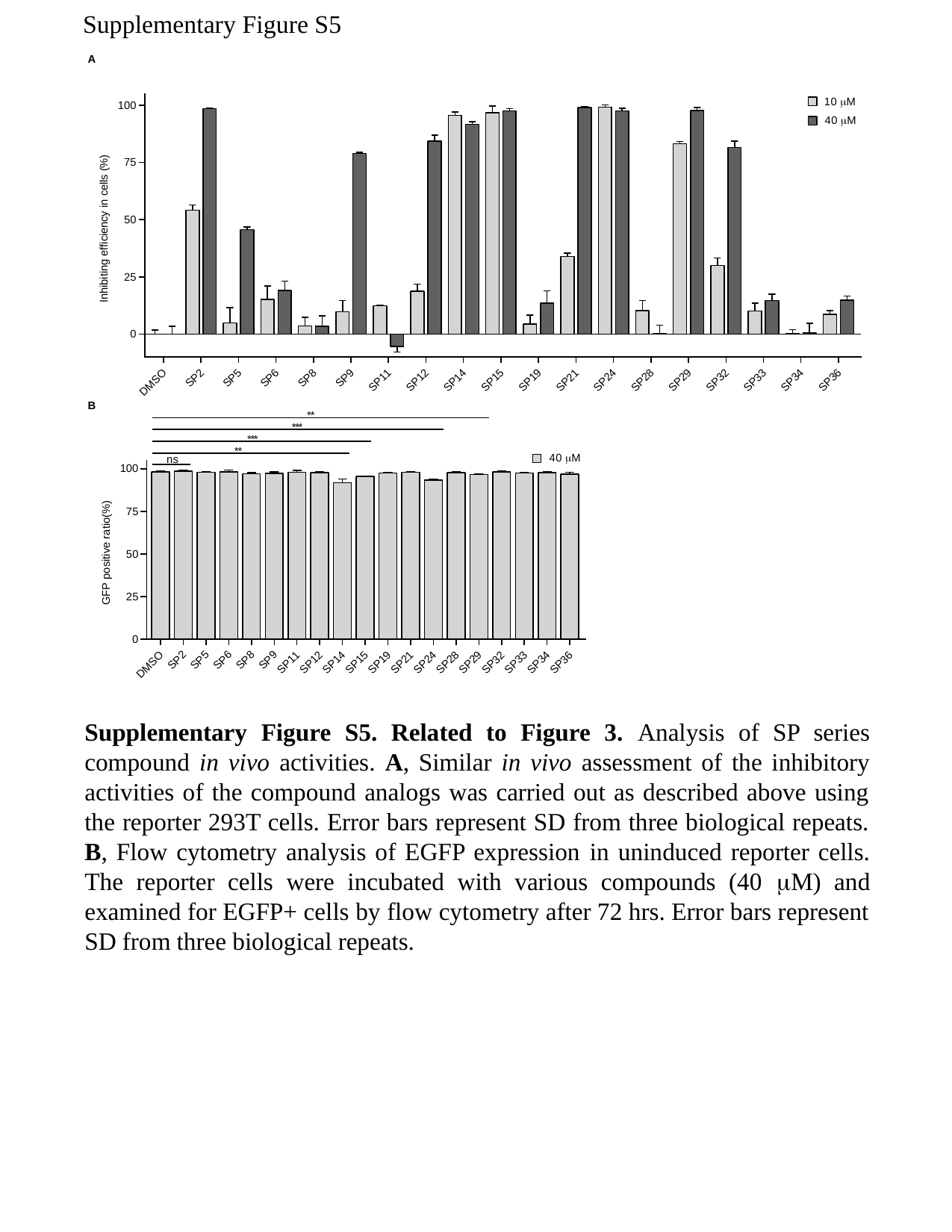

Supplementary Figure S5
A
B
Supplementary Figure S5. Related to Figure 3. Analysis of SP series compound in vivo activities. A, Similar in vivo assessment of the inhibitory activities of the compound analogs was carried out as described above using the reporter 293T cells. Error bars represent SD from three biological repeats. B, Flow cytometry analysis of EGFP expression in uninduced reporter cells. The reporter cells were incubated with various compounds (40 mM) and examined for EGFP+ cells by flow cytometry after 72 hrs. Error bars represent SD from three biological repeats.

## Slide 8
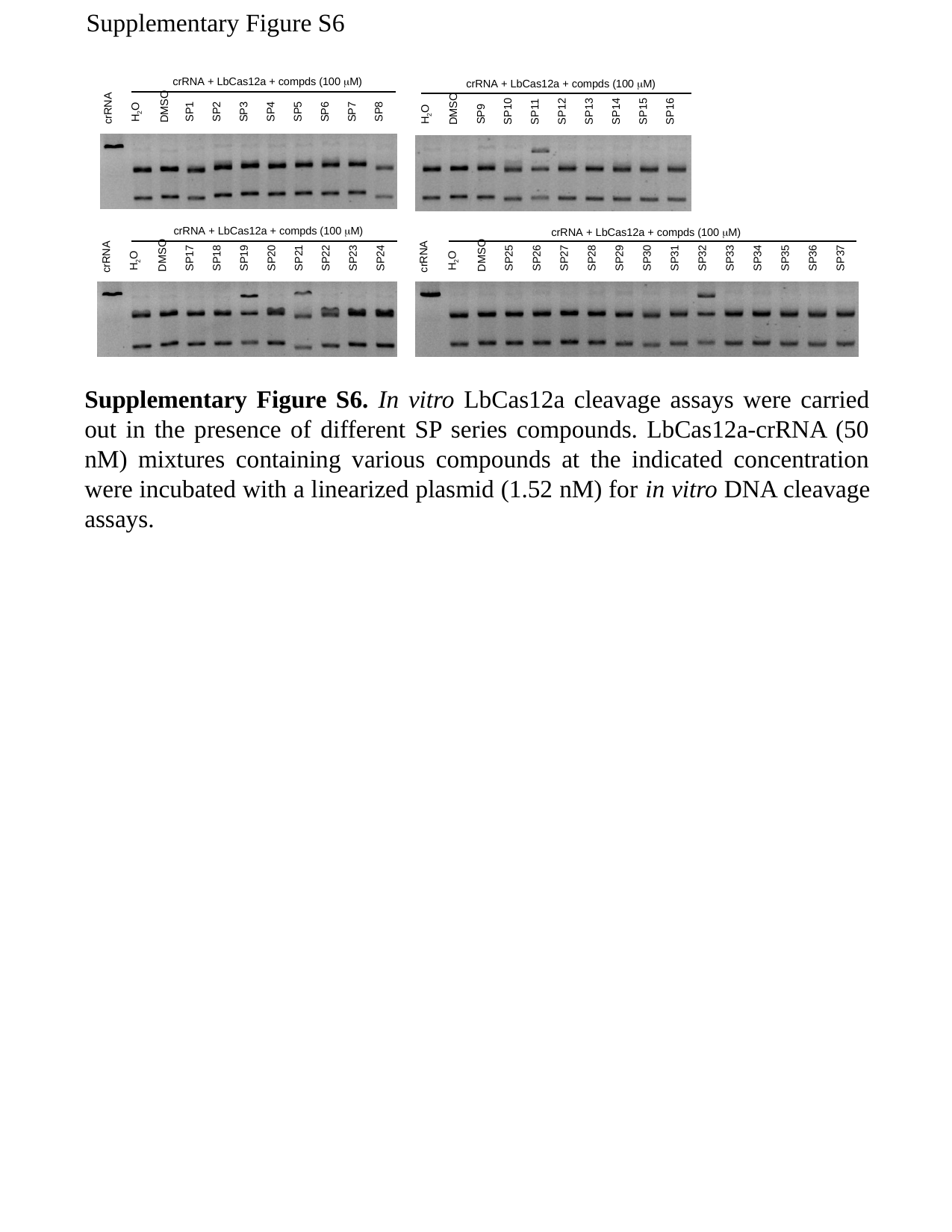

Supplementary Figure S6
crRNA + LbCas12a + compds (100 mM)
DMSO
crRNA
H2O
SP1
SP2
SP3
SP4
SP5
SP6
SP7
SP8
crRNA + LbCas12a + compds (100 mM)
DMSO
SP10
SP11
SP12
SP13
SP14
SP15
SP16
H2O
SP9
crRNA + LbCas12a + compds (100 mM)
DMSO
crRNA
SP17
SP18
SP19
SP20
SP21
SP22
SP23
SP24
H2O
crRNA + LbCas12a + compds (100 mM)
DMSO
crRNA
SP25
SP26
SP27
SP28
SP29
SP30
SP31
SP32
SP33
SP34
SP35
SP36
SP37
H2O
Supplementary Figure S6. In vitro LbCas12a cleavage assays were carried out in the presence of different SP series compounds. LbCas12a-crRNA (50 nM) mixtures containing various compounds at the indicated concentration were incubated with a linearized plasmid (1.52 nM) for in vitro DNA cleavage assays.

## Slide 9
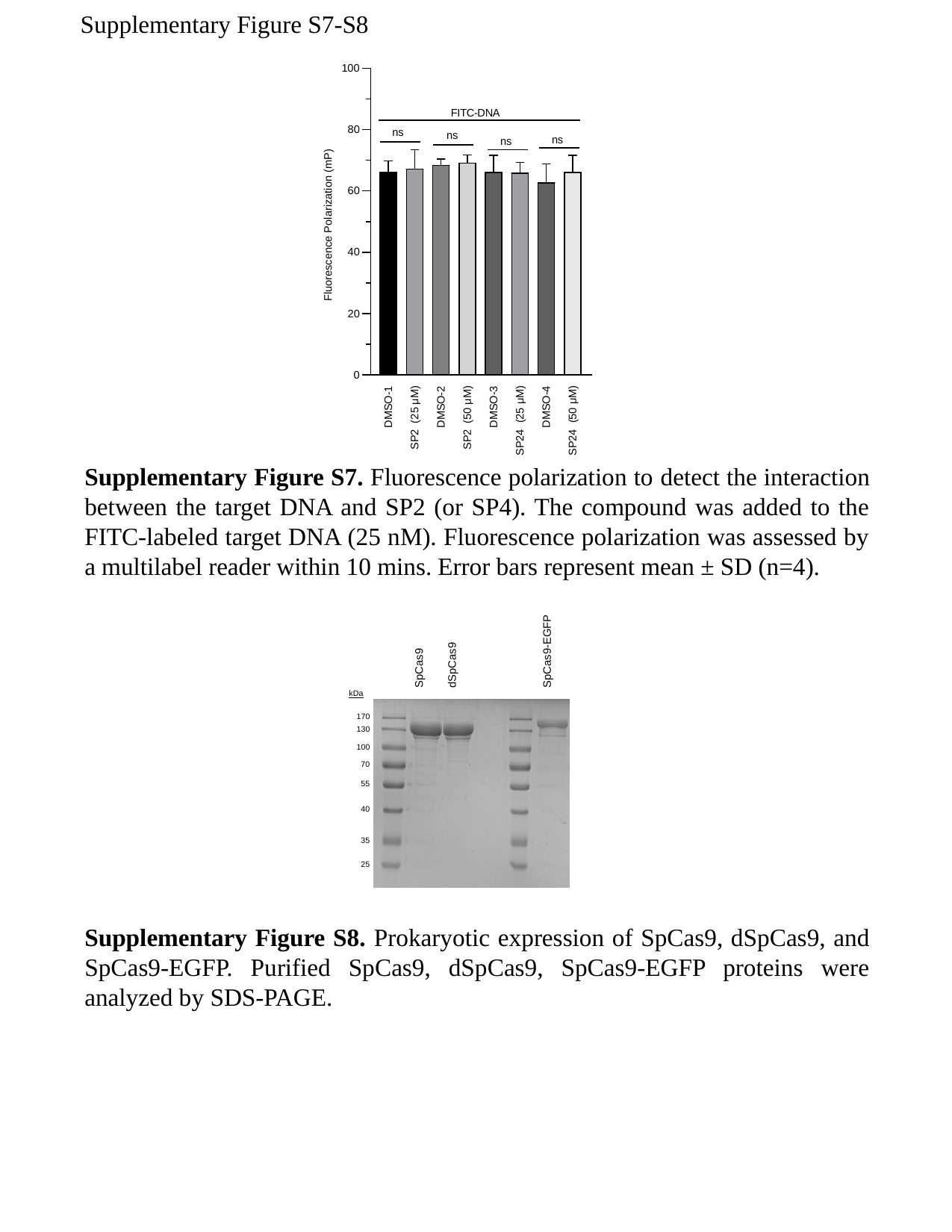

Supplementary Figure S7-S8
Supplementary Figure S7. Fluorescence polarization to detect the interaction between the target DNA and SP2 (or SP4). The compound was added to the FITC-labeled target DNA (25 nM). Fluorescence polarization was assessed by a multilabel reader within 10 mins. Error bars represent mean ± SD (n=4).
SpCas9-EGFP
dSpCas9
SpCas9
kDa
170
130
100
70
55
40
35
25
Supplementary Figure S8. Prokaryotic expression of SpCas9, dSpCas9, and SpCas9-EGFP. Purified SpCas9, dSpCas9, SpCas9-EGFP proteins were analyzed by SDS-PAGE.

## Slide 10
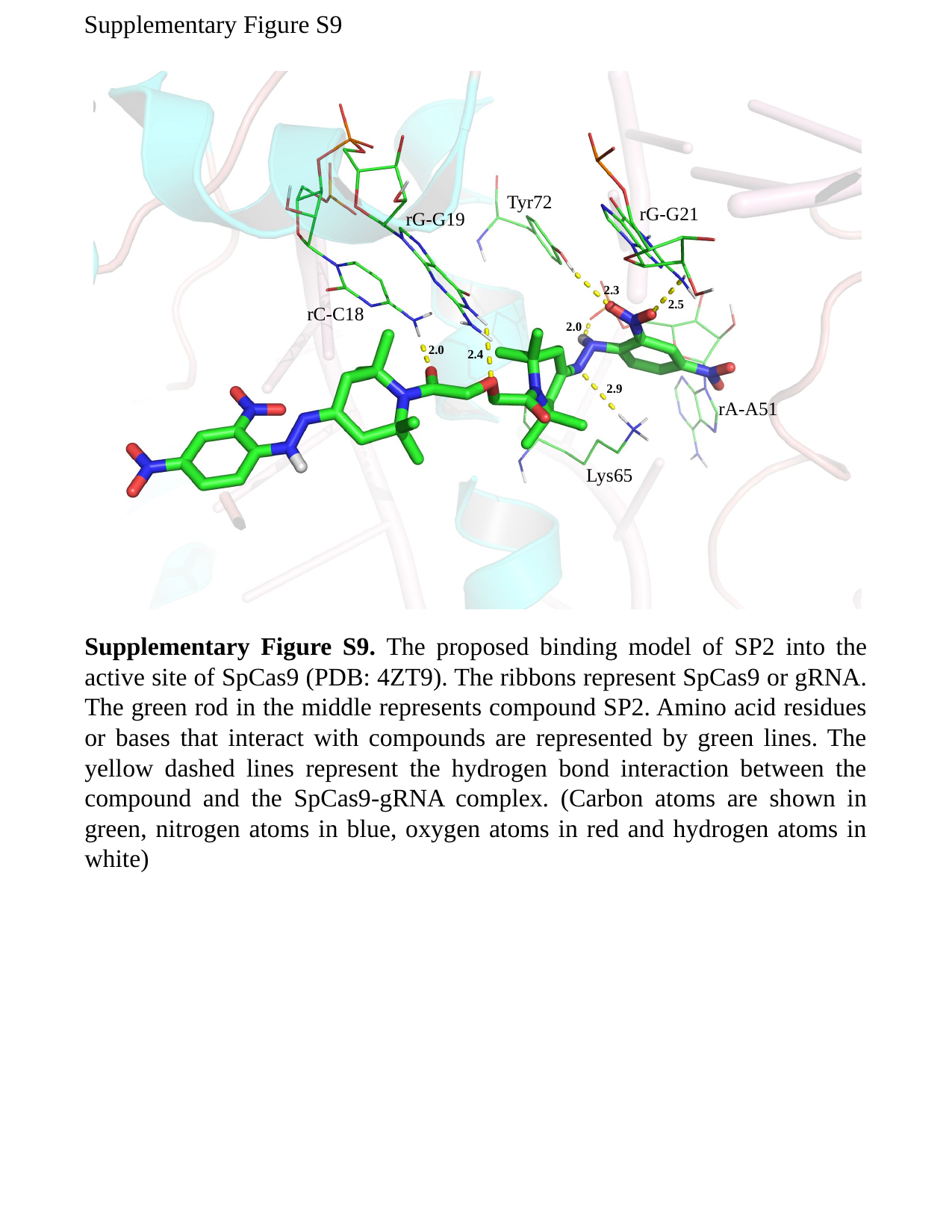

Supplementary Figure S9
Tyr72
rG-G21
rG-G19
2.3
2.5
rC-C18
2.0
2.0
2.4
2.9
rA-A51
Lys65
Supplementary Figure S9. The proposed binding model of SP2 into the active site of SpCas9 (PDB: 4ZT9). The ribbons represent SpCas9 or gRNA. The green rod in the middle represents compound SP2. Amino acid residues or bases that interact with compounds are represented by green lines. The yellow dashed lines represent the hydrogen bond interaction between the compound and the SpCas9-gRNA complex. (Carbon atoms are shown in green, nitrogen atoms in blue, oxygen atoms in red and hydrogen atoms in white)
